# Chromosomal imbalance in pigs showing a syndromic form of cleft palate

**DOI:** 10.1186/s12864-019-5711-4

**Published:** 2019-05-08

**Authors:** Alexander Grahofer, Anna Letko, Irene Monika Häfliger, Vidhya Jagannathan, Alain Ducos, Olivia Richard, Vanessa Peter, Heiko Nathues, Cord Drögemüller

**Affiliations:** 1 Clinic for Swine, Department of Clinical Veterinary Medicine, Vetsuisse Faculty, University of Bern, Bremgartenstrasse 109a, CH-3012 Bern, Switzerland; 2 Institute of Genetics, Department of Clinical Research and Veterinary Public Health, Vetsuisse Faculty, University of Bern, Bremgartenstrasse 109a, CH-3012 Bern, Switzerland; 3 GenPhyse, INRA, INPT, ENVT, Université de Toulouse, 31320 Castanet-Tolosan, France; 4 Institute of Animal Pathology, Department of Infectious Diseases and Pathobiology, Vetsuisse Faculty, University of Bern, Länggassstrasse 122, CH-3012 Bern, Switzerland; 5 Division of Clinical Radiology, Department of Clinical Veterinary Medicine, Vetsuisse Faculty, University of Bern, Länggassstrasse 128, 3012 CH-, Bern, Switzerland

**Keywords:** Pig, Palatoschisis, Teeth, Kidney cysts, Artificial insemination, Malformation, Cryptorchidism, Reciprocal translocation, Whole genome sequencing, SNV array genotyping

## Abstract

**Background:**

Palatoschisis or cleft palate is a known anomaly in pigs resulting in their death. However, little is known about its aetiology. A detailed description of the phenotype was derived from necropsy and by computed tomography revealing that all 20 cases also exhibited hypodontia and renal cysts. Furthermore, a genetic origin was assumed due to dominant inheritance as all 20 recorded cases were confirmed offspring of a single boar.

**Results:**

Single nucleotide variant (SNV) genotyping data were used to map the defect in the porcine genome and led to the detection of a chromosomal imbalance in the affected offspring. Whole genome sequencing of an affected piglet and a normal full sib was used to identify a chromosomal translocation and to fine map the breakpoints in the genome. Finally, we proved that the boar, which sired the malformed piglets, carried a balanced translocation. The detected translocation of Mb-sized segments of chromosome 8 and 14 had not been previously observed during karyotyping. All affected offspring were shown to be carriers of a partial trisomy of chromosome 14 including the *FGFR2* gene, which is associated with various dominant inherited craniofacial dysostosis syndromes in man, and partial monosomy of chromosome 8 containing *MSX1* known to be associated with tooth agenesis and orofacial clefts in other species.

**Conclusions:**

This study illustrates the usefulness of recently established genomic resources in pigs. In this study, the application of genome-wide genotyping and sequencing methods allowed the identification of the responsible boar and the genetic cause of the observed defect. By implementing systematic surveillance, it is possible to identify genetic defects at an early stage and avoid further distribution of congenital disorders.

**Electronic supplementary material:**

The online version of this article (10.1186/s12864-019-5711-4) contains supplementary material, which is available to authorized users.

## Background

Unilateral and bilateral palatoschisis or cleft palate is a well-known congenital anomaly of craniofacial development in several animal species including pigs (OMIA 000197–9823) due to a failure of fusion of the maxillary and medial nasal prominences or between the palatal processes [1–4]. Various chromosomal, Mendelian or teratogenic syndromic and non-syndromic forms of clefts of the palate and/or lip occur in humans and are among the most common birth defects [5] and occur at an approximate rate of 1 in 700 live births [4, 6].

Only limited information regarding the occurrence of palatoschisis or cleft palate in the pig population is available. In one study with a data set of 74,039 live born pigs from nucleus herds, an incidence risk of 0.01 for this congenital disorder was described [7]. Commonly, the affected piglets die within the first days of life due to the aspiration of colostrum into the lungs and consequential associated complications. Little is known about its aetiology, nevertheless, environmental and genetic factors have been considered [1, 8–10]. A known environmental factor, which could induce palatoschisis in piglets, is feeding the sow *Conium maculatum* seed or plant from gestation day 30 to 45 [11]. Moreover, palatoschisis in a piglet was reported after infecting a sow with Classical Swine Fever Virus during gestation [10].

So far, only two studies have determined a precise genetic origin for the occurrence of piglets with cleft palates [1, 8]. The detected chromosomal rearrangement carried by a single sire appeared to be responsible for the malformation observed in the offspring. Indeed in pigs, the kind of balanced reciprocal translocation carried by the boar is relatively common and occurs at an approximate rate of 1 in 200 boars [12–14]. In pigs, reciprocal translocation heterozygotes generally show normal fertility with an appropriate semen quality [15]. However, such a heterozygote carrier of a reciprocal translocation can produce different kinds of gametes, owing to the different segregation mechanisms involved during the first meiotic division [16]. As reported in man and other animal species including pigs, this could lead to a a variable, albeit generally large, proportion of gametes unbalanced for the chromosomes involved in the translocation, which give rise to embryos carrying partial monosomies and trisomies resulting either in embryonic or fetal death, or severely malformed offspring [16, 17].

Since these chromosomal rearrangements have potentially harmful effects in pig breeding programs, their systematic eradication has been advised. Up to now, karyotype analysis of mitogen-stimulated lymphocyte cultures has been the gold standard for the diagnosis of chromosomal abnormalities in pigs [13]. Nonetheless, the evaluation of metaphase spread is a slow, labor-intensive, multi-step process that is difficult to standardize and subject to considerable variability [18]. During the last 10 years, there has been a change due to the development of methods like whole-genome microarray genotyping and/or sequencing which in man have become the diagnostic standard for many chromosomal disorders including craniofacial malformations [19, 20].

The aim of this study was to apply recently improved genomic resources in pigs to evaluate a possible genetic cause for the occurrence of several piglets with palatoschisis in the progeny of a single boar. This study reports detailed phenotypic and molecular investigations, which were performed in order to identify a reciprocal translocation in the sire as the most likely genetic cause for the observed malformations noticed in some of the offspring.

## Methods

### Animals

Due to congenital anomalies in piglets on a satellite farrowing farm in Switzerland, a herd examination was conducted. All sows originated from the same sow pool system and had been artificially inseminated. Adequate data concerning the used boars were not available due to a lack of documentation describing the breeding management system. Therefore, the invoice of the sold semen dose was checked, revealing 6 boars as potential sires of the affected piglets. The sow pool was comprised of Large White x Landrace hybrid females. During gestation, the animals were fed with commercial feed and were provided with fresh water from the public supplier. Water was freely available through a nipple drinker system. The vaccination program of the sow pool system included an immunization against *Erysipelothrix rhusiopathiae* and porcine parvovirus twice a year. Furthermore, twice yearly deworming of all sows was conducted. One week before the expected date of farrowing, the sows were moved to the farrowing unit. This unit was cleaned and disinfected before the animals’ arrival. The pens for free farrowing had a size of 2 × 3 m, with half of the flooring made of concrete, whereas the remaining half was slatted. The sows received a commercial diet two times a day via an automated liquid feeding system. They had unlimited access to water from a bowl drinker. Moreover, the sows received straw as rooting and nest building material. Farrowing induction was not performed in any of the sows. No other relevant medical history of the sows was reported, and further management procedures were analyzed, including gilt management, husbandry and selection of genetics for replacement gilts, revealing no abnormalities. During the examination, 12 piglets (7 male and 5 female) with unilateral or bilateral palatoschisis were noticed in 6 out of 30 litters, which were produced with the semen of the 6 potential boars of different breeds. In these 6 litters a significant decrease (*p*-value: 0.0055, paired t-test; Graph Pad (Graph Pad Software Inc., San Diego, CA)) in total born piglets (14.7 (SD ± 0.9) to 9.7 (SD ± 2.7)) and an increase of stillborn piglets (0.8 (SD ± 0.6) to 1.7(SD ± 1.2)) compared with the former litters was noticed in the affected litters (Table 1). In addition, the stillborn piglets were characterized as antepartum or intrapartum deaths, as described in the literature [21]. Overall, for the 6 examined litters an average of 0.7 piglets per litter were scored for antepartum death and 1.0 piglets per litter scored for intrapartum death. According to good ethical and welfare standards, all 12 affected piglets were euthanized by intravenous injection of an overdose of sodium pentobarbital once blood samples had been obtained for further genetic investigations. In addition, blood samples from the 6 dams and 11 normal littermates, one or two normal siblings per litter, were taken and blood from the six potential boars was provided from the boar stud. Some days after the on-farm examination, skin biopsies of 8 similarly affected piglets of an unspecified number of litters were submitted by the owner. Unfortunately, the owner had no records describing the possible dams. These additional cases were examined neither clinically nor pathologically; however, they were used for the genetic analysis.

**Table 1:** Reproductive parameters of 6 examined litters with malformed piglets in comparison with previous records of the 6 dams

Sow	Litter number (n)	Total born piglets (n/litter) Status quo / before Mean ± SD	Live born piglets (n/litter) Status quo / before Mean ± SD	Dead born piglets (n/litter) Status quo /before Mean ± SD	Palatoschisis (n/litter) Status quo /before Mean
SSC029	2	6.0 / 14.0 ± 0	5.0 / 13.0 ± 0	1 / 1 ± 0	2 / 0
SCC030	6	7.0 / 14.2 ± 0.8	4.0 / 13.0 ± 1.4	3 / 1.2 ± 1.3	1 / 0
SCC031	6	9.0 / 15.6 ± 2.4	8.0 / 15.4 ± 2.5	1 / 0.2 ± 0.5	2 /0
SCC032	7	12.0 / 13.5 ± 1.0	9.0 / 13.3 ± 0.8	3 / 0.2 ± 0.4	6 / 0
SCC033	10	12.0 / 14.9 ± 2.3	10.0 / 14.8 ± 2.4	2 / 0.4 ± 0.5	1 / 0
SSC034	7	12.0 /15.8 ± 1.7	12.0 /14.2 ± 1.9	0 / 1.6 ± 1.9	4 / 0
Average	6.3	9.7 ± 2.7 / 14.7 ± 0.9^a^	8.0 ± 3.0 / 13.9 ± 1.0^b^	1.6 ± 1.2 / 0.8 ± 0.6	2.7 ± 2.0 / 0 ± 0^c^

^a^*p* = 0.0055

^b^*p* = 0.0025

^c^*p* = 0.0210

### Postmortem examination

Twelve malformed piglets belonging to 6 different litters were submitted for full necropsy. Additionally, specimens of the trachea, heart, lung, thyroid gland, liver, kidneys, spleen, pancreas, lymph nodes, umbilicus and upper jaw of four piglets from three different litters were fixed in 4% neutral buffered formalin for histology.

To illustrate the malformations, the head of two affected piglets underwent 16-multidetector-row helical computed tomography (CT) scanning (Brilliance 16, Philips Medical Systems, Eindhoven, The Netherlands). Imaging of the head series were acquired in axial orientation using 120 kV and 180 mAs and were reconstructed using a bone, a soft tissue and a brain algorithm with 1 mm respectively 2 mm slice thickness.

### Genetic analysis

A total of 43 animals (20 cases, 11 normal littermates, 6 dams, and 6 potential sires) were genotyped on GeneSeek GGP Porcine BeadChip containing 50,915 SNVs (Additional file 1). All given SNV positions correspond to the porcine Sscrofa11.1 genome assembly. The PLINK v1.9 software [22] was used to perform basic quality filtering of the dataset and parentage analysis. All genotyped individuals showed call rates > 90% and subsequently 2718 markers with call rates < 90% were excluded. The pruned dataset consisting of 43 animals and 48,197 markers was scanned for Mendelian errors using the “--mendel” option of PLINK v1.9 to reveal any deviations from expected values based on per-individual, per-family, and per-SNV error rates. To confirm the suspected relationships between all animals, especially the suspected boars, the option “--genome” was used to determine the relationship based on the genomic information. This resulted in the exclusion of 5 boars as potential sires and confirmed a single boar (SSC040) as sire of all 31 sampled piglets. Subsequently, a combination of two SNV array measures, the log R ratio (LRR) and the B allele frequency (BAF), were used to detect possible copy number variants (CNV) in the affected animals. The R is a normalized intensity value that portrays the relative amount of each SNV across the chromosome compared to diploid individuals [23]. The BAF, where the B allele can have values of 1 (BB), 0.5 (AB) and 0 (AA) in a diploid individual, was explored to identify the origin of a CNV. Considering that uniparental disomy can occur in the genome due to rescue mechanisms [23], SNVs in the regions of interest were selected, which had a BAF 0 for the sire and 1 for the dam or vice versa. With those SNVs, where each one parent is homozygous for alternative alleles, it was possible to identify from which parent the additional allele came from.

### Whole genome sequencing

In order to localize the chromosomal breakpoints precisely, whole genome sequences of one affected piglet (Additional file 1: animal SSC006) and one non-affected littermate (Additional file 1: animal SSC028) were produced. The Illumina HiSeq3000 was used to produce 175,383,409 (affected piglet) and 117,590,672 (normal piglet) paired-end reads of 2 × 150 bp length. The reads were mapped to the pig reference genome using the Burrows-Wheeler Aligner version 0.7.5.a [24] with default settings. After sorting the mapped reads by the coordinates of the sequence with Picard tools, the read duplicates were also labelled with Picard tools version 1.8 (http://sourceforge.net/projects/picard/). The Genome Analysis Tool Kit [GATK version v3.6, (https://www.ncbi.nlm.nih.gov/pubmed/20644199)] was used to perform local realignment and to produce a cleaned BAM file. The reference genome used is the Sscrofa11.1 and its annotation from NCBI release 106 (https://www.ncbi.nlm.nih.gov/genome/annotation_euk/Sus_scrofa/106/). Furthermore, to map the breakpoints of the structural variants the integrative genome viewer [25] was used. The chimeric read-pairs in which both ends mapped to different chromosomes were extracted using samtools [26] and the soft clipped part of the reads bridging the breakpoints were searched in the current pig genome assembly by BLAST (https://blast.ncbi.nlm.nih.gov/Blast.cgi?PAGE_TYPE=BlastSearch&PROG_DEF=blastn&BLAST_SPEC=Assembly&ASSEMBLY_NAME=GCF_000003025.6).

To calculate the coverage, a sliding window approach was used where the window size was 10 kb and was moved for half the window size. Using the function bedcov of the program samtools [26], the output generated was the number of reads within each specified window. Therefore, we further multiplied it by the length of the reads (150 bp) and divided it by the length of the window in order to receive the average coverage per base in each window. The circos plot was created using OmicCircos package [27].

### Mapping breakpoint regions by PCR and sanger sequencing

Genomic DNA sequences flanking the putative breakpoint regions were extracted from Sscrofa11.1. Validation primers were designed using Primer3 software (http://bioinfo.ut.ee/primer3-0.4.0/) using standard parameters primers (Additional file 2). The putative fragments were amplified purified and sequenced using an ABI 3730 DNA analyzer (Thermofisher). The multiplex PCR products of various lengths were separated by capillary gel electrophoresis on the Fragment Analyzer Automated CE System (Advanced Analytical Technologies) to infer the different individual karyotypes.

### Chromosomal analyses

Giemsa-trypsin G (GTG)-banding karyotype of the sire was established by classical protocols [28] used within the chromosomal control program carried out for all boars intended for usage in artificial insemination in Switzerland.

## Results

**Fig. 1:**
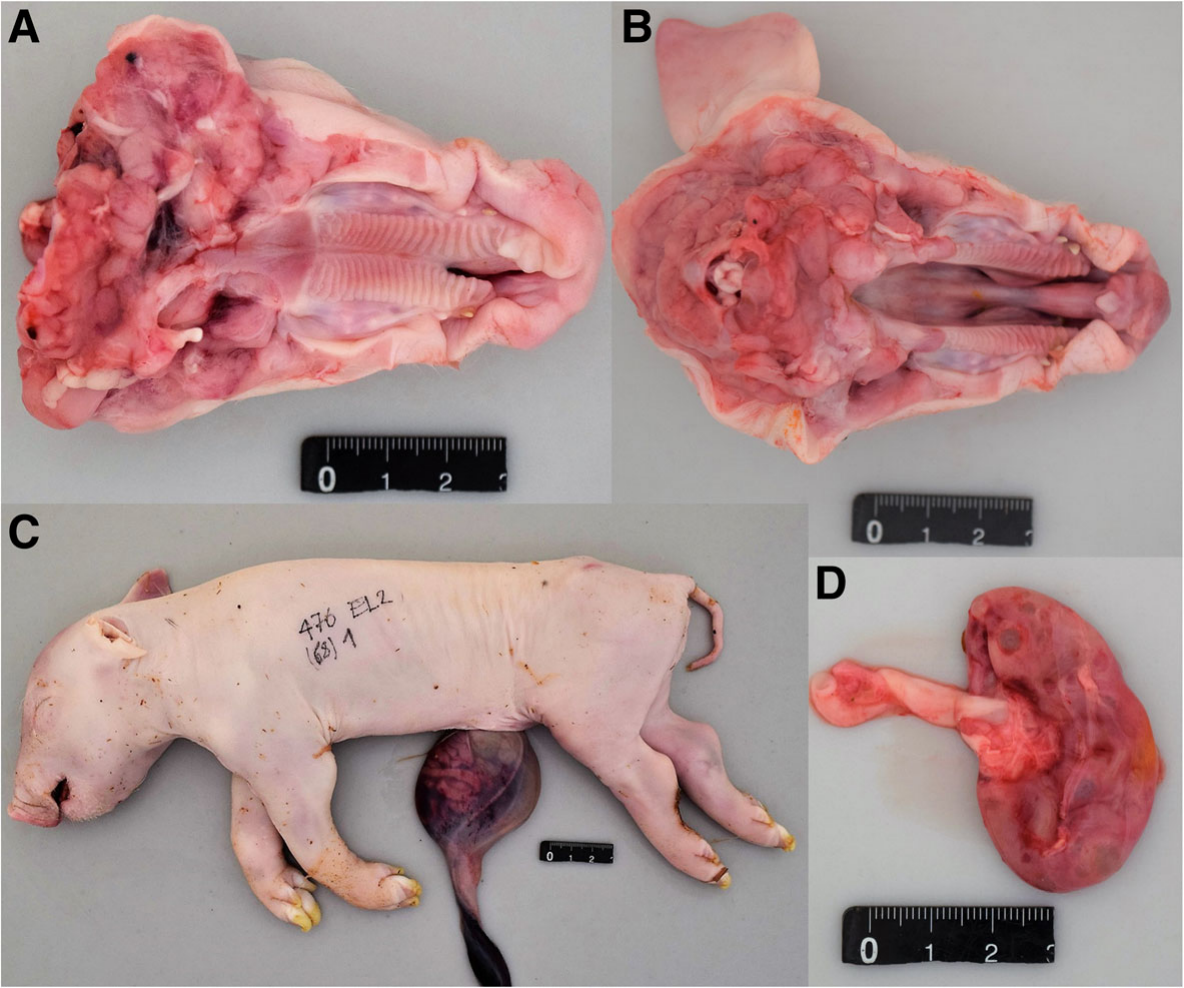
Gross morphological findings in cleft palate-affected newborn piglets. An example for unilateral (**a**) and bilateral (**b**) palatoschisis is shown. Note the hernia umbilicalis (**c**) which occurred in some of the affected piglets. All affected piglets showed multiple renal cysts (**d**)

**Fig. 2:**
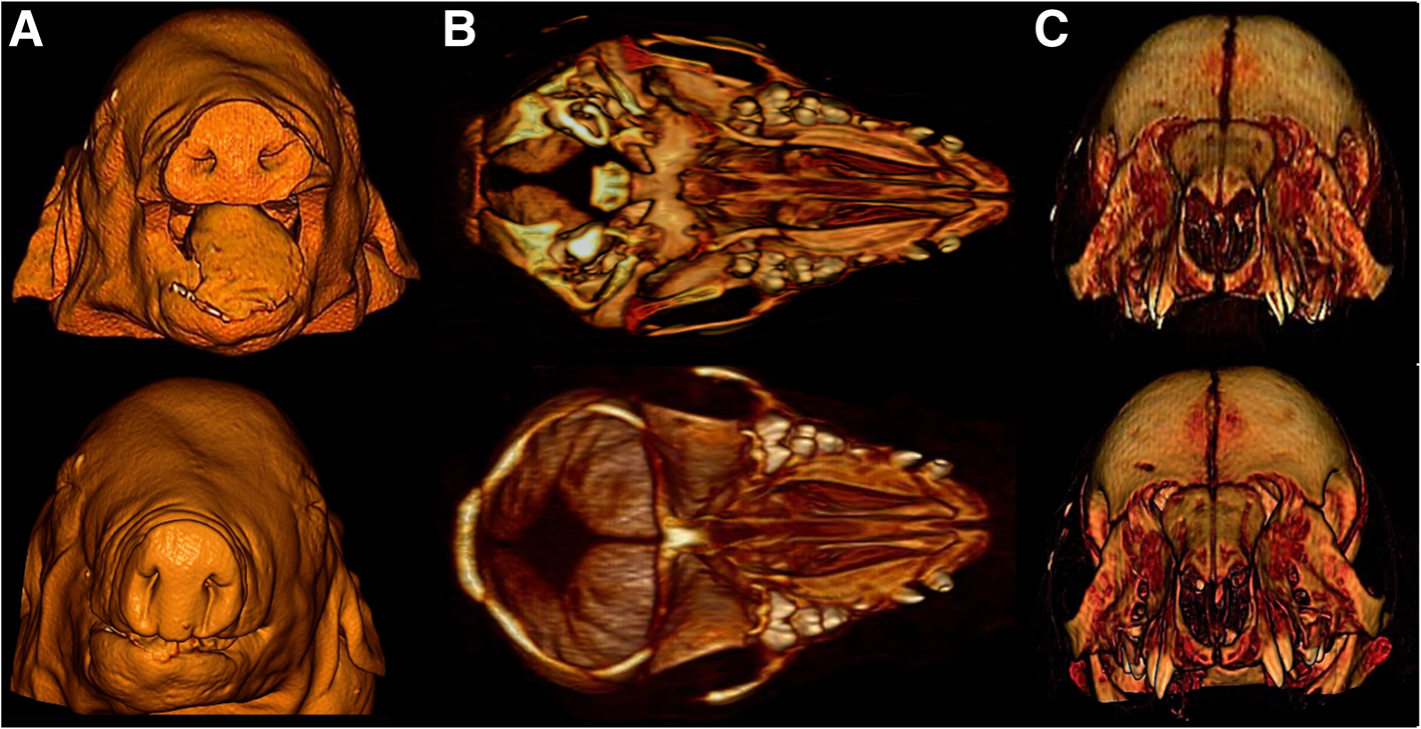
Computed tomography images of two affected piglets with cleft palate. **a**: The nostrils of one case (top) were also affected while the snout of the second case (below) looked normal. Note the absence of the maxillary first and second incisive (**b**), as well as the absence of the hard and soft palate in both affected piglets (**c**)

In the macroscopic examination, all 12 piglets from 6 litters displayed variable stages of malformation of the snout, the dorsal lip and the hard and the soft palate (Figs. 1 and 2; Additional file 3). Eight piglets had a bilateral defect in closure of the snout, the dorsal lip, the hard and the soft palate (cheilognatopalatoschisis) (Figs. 1 and 2). Three piglets displayed bilateral defects in closure of the hard and soft palate (palatoschisis). Two piglets displayed either a unilateral defect in closure of the snout and dorsal lip (cheilognatoschisis) or unilateral cheilognatopalatoschisis.

**Fig. 3:**
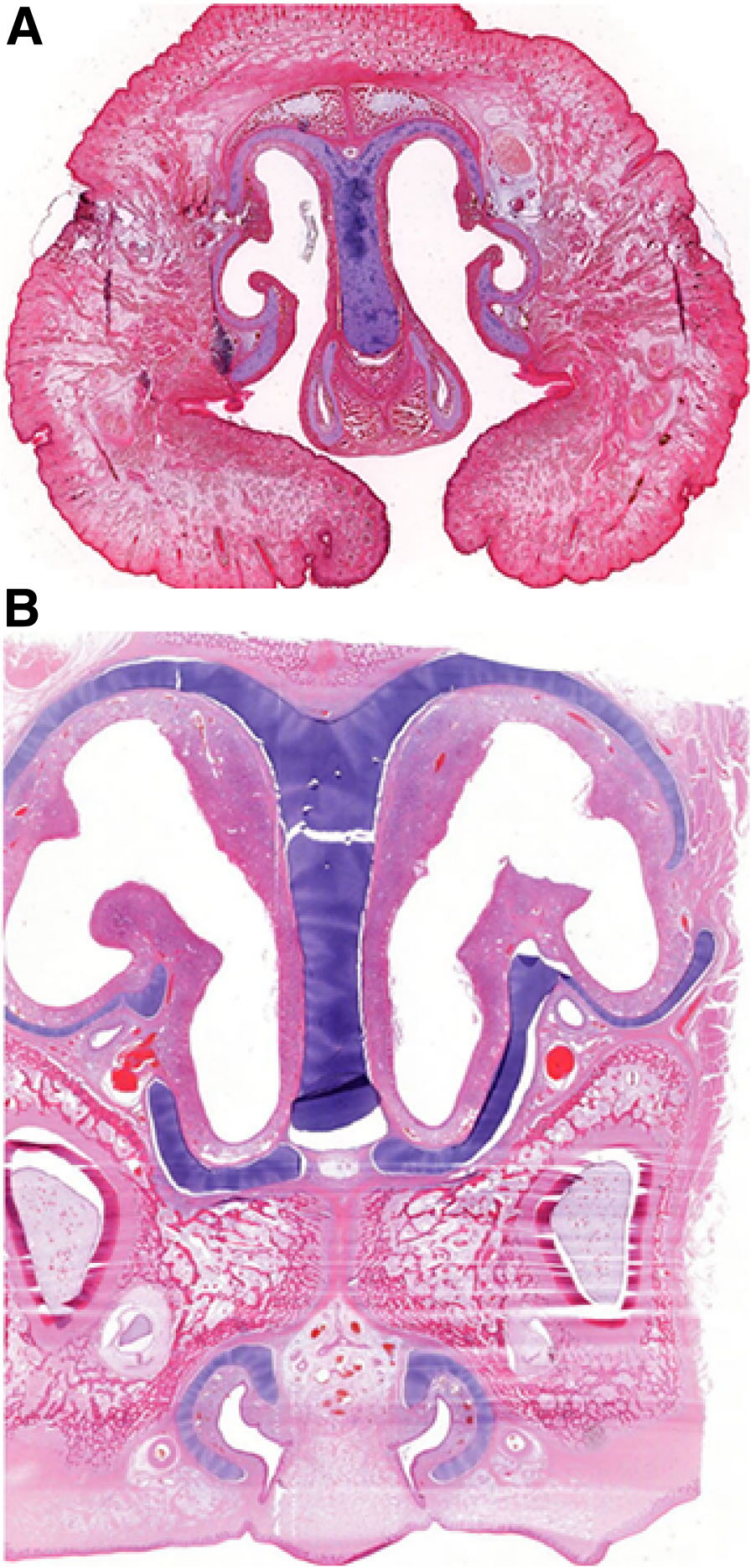
Histopathological comparison of the hard palate of an affected piglet (**a**) and an age-matched control (**b**). Note that in the affected piglet the frontal process failed to fuse with the maxillary process

Additionally, all examined affected animals showed renal cysts, all male piglets showed cryptorchism, and three piglets had umbilical herniation with protrusion of small intestine (Fig. 1 c, d; Additional file 3). In the histopathological examination, no further changes were diagnosed in the aforementioned organs (Fig. 3).

**Fig. 4:**
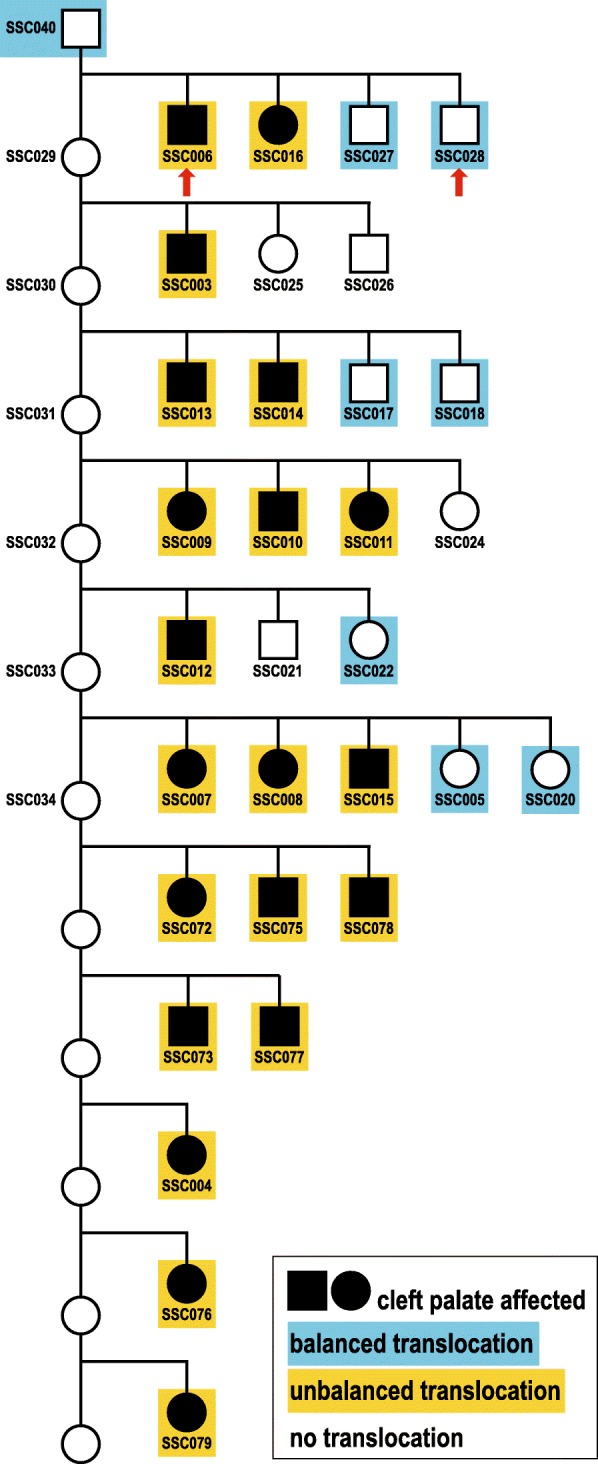
Cleft palate segregation in the progeny of a heterozygous carrier boar with a balanced reciprocal translocation. Note that all piglets tested for the presence of the unbalanced translocation were affected (shown in yellow). Piglets that inherited the balanced reciprocal translocation are shown in blue. Palatoschisis-affected piglets are depicted as filled symbols, normal animals as white symbols. Females are shown as circles, males as squares. All 38 numbered animals were available for SNV array genotyping. The whole genomes of the two piglets indicated with red arrows were sequenced

Utilizing the single nucleotide variant (SNV) array genotyping data, a pedigree of all animals revealing a single Piétrain boar as the only plausible sire of all 20 affected piglets was constructed (Fig. 4). The maternity status of the sampled 6 sows in respect of the examined 12 affected and 11 non-affected piglets was confirmed and the later submitted 8 affected piglets were assigned to 5 different litters. For the 6 examined litters with genotyped parents and offspring, the SNV genotypes were analyzed for Mendelian errors. A total of 3178 Mendelian errors were obtained, of which 1967 were clustered on the proximal 25 Mb of chromosome 8, while the rest were randomly distributed across the genome (Additional file 4). Across the entire chromosome 8, about 14.4% of markers contained errors, while it was on average only 1.5% of markers on the other autosomes. Interestingly, we observed a strikingly higher number of paternal errors in the affected piglets compared to normal littermates. For the 6 examined litters we observed on average 183.3 paternal errors across the genome in the affected, but only 17.2 in the unaffected piglets.

**Fig. 5:**
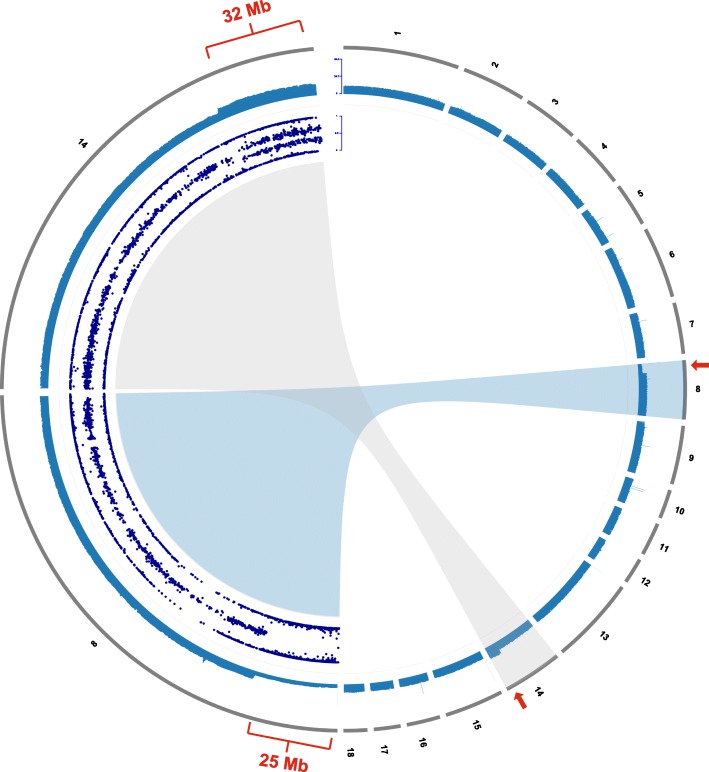
Autosome representation of cleft palate-affected piglets showing an unbalanced reciprocal translocation t (8,14). Note the red marks highlighting the partial monosomy of chromosome 8 and the partial trisomy of chromosome 14. The 18 porcine autosomes are depicted in the right half of the circle as grey bars, and the average sequence depth for the 500 kb windows of a sequenced affected piglet is shown in blue below. The left half of the circle displays a close-up view of chromosome 8 and 14. The sequence coverage plot is shown accordingly while the inner circular track shows the B allele frequencies (BAF) at genotyped SNVs of the affected piglet on chromosome 8 and 14 in dark-blue. Note the clusters of BAF around 0.33 and 0.66 in the distal 32 Mb of chromosome 14 indicating the trisomy, and the missing BAF of 0.5 in the proximal 25 Mb of chromosome 8 indicating the monosomy

To detect the possible presence of a chromosomal imbalance explaining these clustered Mendelian errors the log R ratio (LRR) was subsequently analyzed. All affected piglets showed a drop of LRR in the proximal region (0–25 Mb) of chromosome 8 and, surprisingly, also an increase in the distal region (109–142 Mb) of chromosome 14 (Additional file 5). Therefore, the B allele frequency (BAF) was then investigated as a measure of allelic imbalance to detect any aberration of the expected values of 0, 0.5, and 1 representing normal diploid genotypes. Interestingly we observed in all affected individuals within the 32 Mb distal region of chromosome 14, two clusters of BAF around 0.33 and 0.66 deviating from the expected 0.5 value (Fig. 5; Additional file 6). This indicated the presence of three copies for this genome segment, whereas in the proximal region of chromosome 8 the BAF of 0.5 does not occur, which indicates monosomy. Based on SNVs that had alternative homozygous genotypes in the parents we detected that the obvious imbalance was inherited from the father (Additional file 6). In all affected piglets, the underrepresentation of paternal alleles on chromosome 8 and the overrepresentation of paternal alleles on chromosome 14 indicated the presence of an unbalanced reciprocal translocation inherited from the father (Additional file 6).

**Fig. 6:**
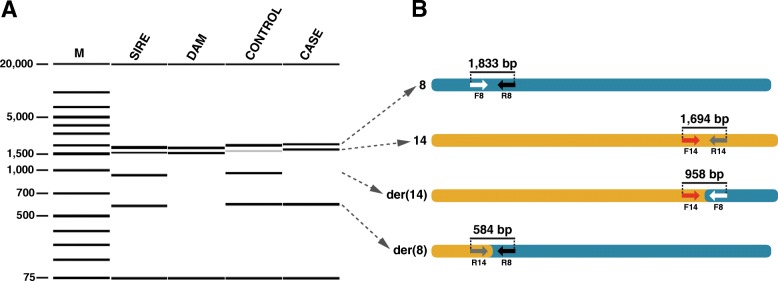
PCR-based genotyping of the detected reciprocal translocation**.** Visualization of PCR products obtained from normal chromosomes (8 and 14) and two different derivative chromosomes (der (14) and der (8)). Note that the der (14) junction fragment is absent in this case and its dam whereas the der (8) junction fragment is present in both offspring and their sire. M: DNA ladder

Whole genome sequencing (WGS) of one affected piglet at ~13x coverage and one normal littermate at ~16x coverage showed a reduced sequence coverage in the proximal 25 Mb of chromosome 8 and an increased coverage in the distal 32 Mb of chromosome 14 in the affected animal (Fig. 5). Visual inspection of discordant pair-end sequence reads aligning on chromosomes 8 and 14 confirmed the suspected reciprocal translocation (Additional file 7). Based on the Sanger sequencing results, we mapped and analyzed the breakpoint region of the t (8,14) translocation (Fig. 6). Both breakpoints (chr8:25,855,619 and chr14:109,710,060) were perfectly balanced without any loss or gain of sequence on either derivative chromosome. Multiplex PCR showed that the boar carried a balanced translocation while the tested 6 dams had exclusively normal chromosomes (Figs. 4 and 6; Additional file 1). Cytogenetic karyotyping of the boar revealed no microscopically visible indication for the detected reciprocal translocation (Additional file 8). Among the tested 12 unaffected (and genomically balanced) littermates, seven inherited both derivative chromosomes of the boar (der (8) + der (14)), whereas five others received the normal (8 + 14) paternal chromosomes (Fig. 4; Additional file 1). Furthermore, PCR-based genotyping confirmed that the unbalanced translocation was present in all 20 affected piglets with a haploinsufficiency of ~ 25 Mb of chromosome 8 and a partial trisomy of ~ 30 Mb of chromosome 14 (Fig. 4; Additional file 1).

## Discussion

A novel congenital syndrome was recognized in the progeny of a single artificial insemination sire. The gross, microscopic and radiographic lesions in the examined piglets of the Piétrain boar are consistent with congenital palatoschisis, a condition also referred to as cleft palate. To date, only a few studies have described and characterized palatoschisis in piglets [1, 2, 11]. Unfortunately affected animals are mostly remain unreported, some farmers do not discover the anomaly and others might not report congenital disorders in their breeding animals due to possible negative impact of the reputation of their breeding herds. Thus, the incidence of palatoschisis is most likely to be underestimated in the literature. In all piglets affected with cleft lips, a heavily malformed snout was detected. The development of the rostrum is closely connected with lip formation [2]. To the author’s knowledge, this report is the first describing hypodontia in palatoschisis-affected piglets. Normally, piglets are born with 28 teeth, including three incisors, one canine tooth and three premolars per jaw half [29]. Tooth development is genetically regulated and is a result of a series of inductive, sequential, and reciprocal interactions between the ectoderm and the subjacent mesenchyme [30]. Aberrations in the development process can lead to permanent morphologic consequences of the teeth. In humans, palatoschisis is often associated with numerous dental defects such as congenitally missing teeth [31, 32].

In this case, an adequate documentation of the breeding management was missing; therefore, the first step of the genetic analysis was to clarify the paternity of affected piglets. Medium density SNV genotyping of piglets, dams and potential sires was performed revealing a single boar as the common father in all cases. As time is always an important factor in the spreading of an inherited congenital defect, an appropriate breeding management including the in-depth exploration of genomic information would have had identified the responsible boar earlier and action could have been undertaken faster (e.g. culling). In this study, the offspring were produced after mating of two breeds, suggesting that the causative mutation is not a rare recessive variant.

Chromosome abnormalities are associated with reduced reproductive performance [33]. In humans and mice, reciprocal chromosome exchanges are known to cause disturbances during gametogenesis [34]. Boars, which carry reciprocal chromosomal translocations negatively, influence the reproductive performance in sow herds and the viability of their offspring. Reciprocal chromosomal translocations are the most common structural chromosomal rearrangements in domestic pigs [15]. Due to the extensive use of artificial insemination in the pig industry and the fact that up to 80 semen doses are produced from every boar’s ejaculate, semen doses of one single boar can become widely distributed in the sow population in short time. Therefore, a single carrier boar can cause enormous economic losses in pig production, due to fewer viable piglets. In addition, presence of a congenital disorder produces animal welfare issues for the affected animals. Therefore, the detection of a reciprocal translocation in a boar at an early stage is essential. In this report, a significant reduction in litter size of the affected litters was expected from the identified sire, and the number of affected piglets varied between one and six piglets per litter. This is in line with another report where a single boar with a balanced reciprocal translocation sired approximately 100 litters. The number of affected piglets per litter varied between zero and eight, and nearly 400 piglets were born with palatoschisis [8]. The karyotype of the boar, which sired the malformed piglets, presented in this study had been evaluated within the chromosomal control program carried out for all boars intended for usage in artificial insemination in Switzerland. Although the translocated segments of chromosome 8 and 14 differed in size by 7 Mb, this reciprocal translocation, confirmed by PCR to be present in the boar, could obviously not be detected with regular microscopic resolution. The 7 Mb represents a value which is close to the average size of one band on a GTG-banding karyotype, i.e. close to the resolution limit of classical cytogenetics techniques. This highlights the importance but also the limitations of classical cytogenetic analysis. This study showed for the first time in pigs, that SNV array genotyping data can provide superior resolution in comparison to metaphase karyotype analysis to detect sub-microscopic balanced translocations. As is the case in man, cost-effective SNV array genotyping with higher potential accuracy could be applied to detect numeric abnormality of chromosome segments in domestic animals [35]. In addition, we confirmed that whole genome sequencing in combination with paired-end mapping methods allows the detection of breakpoints at single base pair resolution [36]. Such studies in pigs have recently become possible owing to advances in genotyping and sequencing methods [37] in combination with the largely improved genomic resources [38]. Thanks to the combination of SNV array data and whole genome sequences, we were able to characterize the precise nature of the t (8,14) translocation. Subsequent analyses of LRR and BAF provided evidence for the presence of a chromosomal imbalance in the affected piglets and furthermore confirmed the paternal origin of the unbalanced translocation. We observed that from the four possible gametes produced by the heterozygous carrier of the reciprocal translocation (via alternate and adjacent-1 segregation mechanisms), only three occurred in the offspring. Besides the normal gametes and unbalanced gametes (containing one normal chromosome 14 and a rearranged der (8) chromosome) observed in the affected piglets, only balanced gametes carrying the two rearranged chromosomes were detected in normal offspring. Therefore, we assume that the fourth kind of gametes containing one normal chromosome 8 and a shorter chromosome 14 and an additional part of chromosome 8 (rearranged der (14) chromosome), as well as gametes produced by adjacent-2, 3:1 or 4:0 segregation mechanisms, might result in early embryonic death.

The results presented here strongly resemble that reported in piglets with cleft palates from the progeny of a boar with a constitutional balanced reciprocal translocation [1]. Chromosomal analyses of five affected piglets showed that they all had an identical unbalanced karyotype with partial monosomy of chromosome 16 and partial trisomy of chromosome 3, whereas the normal piglets in the litters had balanced karyotypes. The authors hypothesized that the congenital malformation observed in the piglets with an unbalanced karyotype was probably the result of the presence of excess genes and/or the result of the presence of only one copy of genes. Since in man various chromosomal and Mendelian syndromic and non-syndromic forms of clefts of the palate and/or lip are associated with single genes [5], one could speculate those candidate genes which map to the porcine genomic regions concerned with the chromosomal imbalance are causing the congenital malformation. Three candidate genes (*FGF8*, *VAX1 FGFR2*) map to the 32 Mb segment of chromosome 14 showing the trisomy in the affected piglets, and a single candidate gene (*MSX1*) maps to the haploinsufficient 25 Mb segment of chromosome 8. Interestingly, heterozygous variants of *MSX1* (OMIM 106600) have been identified in human patients with various rare autosomal dominant conditions characterized by tooth agenesis with or without orofacial cleft [39]. Therefore, it seems to be likely that the detected *MSX1* haploinsufficiency plays a role in the occurrence of the cleft palate and hypodontia phenotype in the affected piglets. Nonetheless, this syndromic phenotype is most likely to be also influenced by possible dosage effects of other genes e.g. *FGFR2*, a member of the fibroblast growth factor receptor family, as variants in this gene are associated with different autosomal dominant inherited craniosynostosis syndromes (OMIM 176943). Interestingly, a recently discovered de novo missense variant of bovine *FGFR2* causes facial dysplasia syndrome with palatoschisis in Holstein cattle [40].

## Conclusions

Systematic surveillance in breeding programs, including whole genome analysis, is needed to identify genetic defects as early as possible in order to avoid further losses. Thus, the detection and characterization of chromosomal imbalances are especially important in pigs as they are reported to show a high number of reciprocal translocations and other structural constitutional rearrangements. This study illustrates the usefulness of recently established genomic resources in pigs. In this study, the application of genome-wide genotyping and sequencing methods allowed the identification of the responsible boar and the genetic cause of the observed defect.

## Additional files

Additional file 1: Phenotype records and translocation genotypes of 43 pigs. (PDF 221 kb)

Additional file 2: Primers used for genotyping. (PDF 153 kb)

Additional file 3: Detailed phenotype records of 12 affected piglets. (PDF 3111 kb)

Additional file 4: Mendelian error mapping. The genomic position of the 3178 SNVs showing Mendelian errors is shown along the porcine chromosomes (above). Note the clustering of the errors in the proximal 25 Mb of chromosome 8 (below). (PDF 1105 kb)

Additional file 5: Examples of log R ratio (LRR) plots. The LRR is shown along the chromosomes for all SNVs on the array. Examples for 4 animals are shown for chromosomes 8 and 14. Note that the LRR drop in the proximal region of chromosome 8 and the increase in the distal part of chromosome 14 in the affected piglet (shown in red). (PDF 2021 kb)

Additional file 6: Segregation of SNV alleles. In all 6 examined families for both parents alternative homozygous SNVs (red/blue) were selected to determine their inheritance pattern. Note that in all affected piglets paternal homozygous SNVs show an underrepresentation on chromosome 8 and an overrepresentation on chromosome 14. For SNVs where the dams were homozygous for the alternative allele, an opposite segregation could be observed. (PDF 2183 kb)

Additional file 7: IGV snapshot indicating the translocation. Note the reduced coverage on chromosome 8 and the increased coverage on chromosome 14 in the affected piglet. Paired-end sequence reads mapping on two different chromosomes are displayed in different colors. (PDF 2757 kb)

Additional file 8: Karyotype of the boar: 38, XY, t (8,14). Note that the two affected chromosomes show an unobvious abnormal banding profile (above). A comparison of the chromosomes 8 (top) and 14 (bottom) from 8 different cells of the boar revealed no microscopically visible difference (below). (PDF 1136 kb)

## Abbreviations

BAF: B allele frequency
BAM: Binary version of a sequence alignment/map (SAM) file
BLAST: Basic local alignment search tool
bp: Base pairs
CNV: Copy number variant
kb: Kilo base pairs
LRR: log R ratio
Mb: Mega base pairs
OMIA: Online mendelian inheritance in animals
PCR: Polymerase chain reaction
SNV: Single nucleotide variant
