# Supplementary material for: Chromosomal imbalance in pigs showing a syndromic form of cleft palate
Source: BMC Genomics. 2019 May 8;20:349. doi: 10.1186/s12864-019-5711-4 (PMC6505205; doi:10.1186/s12864-019-5711-4)
Supplement: Supplementary file 1 — Phenotype records and translocation genotypes of 43 pigs. (PDF 221 kb) [file 12864_2019_5711_MOESM1_ESM.pdf]

Additional file 1 Phenotype records and translocation genotypes of 43 pigs.

Lab ID	Father	Mother	Sex	Sample	Status	Maxillar malformation	Umbilicus	Kidney	Testis	WGS	der(14)	der(8)
SSC006	SSC040	SSC029	male	blood	case	bilateral palatoschisis (hard and soft palate)	normal	multiple cysts	unilateral cryptorchid	WGS	-	+
SSC016	SSC040	SSC029	female	blood	case	bilateral palatoschisis (hard and soft palate)	normal	multiple cysts			-	+
SSC027	SSC040	SSC029	male	blood	normal						+	+
SSC028	SSC040	SSC029	male	blood	normal					WGS	+	+
SSC003	SSC040	SSC030	male	blood	case	bilateral palatoschisis (hard and soft palate)	umbilical hernia	multiple cysts	bilateral cryptorchid		-	+
SSC025	SSC040	SSC030	female	blood	normal						-	-
SSC026	SSC040	SSC030	male	blood	normal						-	-
SSC013	SSC040	SSC031	male	blood	case	bilateral palatoschisis (hard and soft palate)	umbilical hernia	multiple cysts	unilateral cryptorchid		-	+
SSC014	SSC040	SSC031	male	blood	case	bilateral cheilognathopalatoschisis (hard and soft palate)	normal	multiple cysts	unilateral cryptorchid		-	+
SSC017	SSC040	SSC031	male	blood	normal						+	+
SSC018	SSC040	SSC031	male	blood	normal						+	+
SSC009	SSC040	SSC032	female	blood	case	bilateral cheilognathopalatoschisis (hard and soft palate)	normal	multiple cysts			-	+
SSC010	SSC040	SSC032	male	blood	case	bilateral cheilognathopalatoschisis (hard and soft palate)	normal	multiple cysts	bilateral cryptorchid		-	+
SSC011	SSC040	SSC032	female	blood	case	bilateral cheilognathopalatoschisis (2/5 of the hard palate)	normal	multiple cysts	unilateral cryptorchid		-	+
SSC024	SSC040	SSC032	female	blood	normal						-	-
SSC012	SSC040	SSC033	male	blood	case	bilateral cheilognathopalatoschisis (hard and soft palate)	normal	multiple cysts	bilateral cryptorchid		-	+
SSC021	SSC040	SSC033	male	blood	normal						-	-
SSC022	SSC040	SSC033	female	blood	normal						+	+
SSC005	SSC040	SSC034	female	blood	normal						+	+
SSC007	SSC040	SSC034	female	blood	case	bilateral cheilognathopalatoschisis (hard and soft palate)	umbilical hernia	multiple cysts			-	+
SSC008	SSC040	SSC034	female	blood	case	bilateral cheilognathopalatoschisis (hard and soft palate)	normal	multiple cysts			-	+
SSC015	SSC040	SSC034	male	blood	case	bilateral cheilognathopalatoschisis (2/5 of the hard palate)	normal	multiple cysts	unilateral cryptorchid		-	+
SSC020	SSC040	SSC034	female	blood	normal						+	+
SSC072	SSC040	unknown dam1	female	tissue	case	palatoschisis, owner reported	unknown	unknown			-	+
SSC075	SSC040	unknown dam1	male	tissue	case	palatoschisis, owner reported	unknown	unknown	unknown		-	+
SSC078	SSC040	unknown dam1	male	tissue	case	palatoschisis, owner reported	unknown	unknown	unknown		-	+
SSC073	SSC040	unknown dam2	male	tissue	case	palatoschisis, owner reported	unknown	unknown	unknown		-	+
SSC077	SSC040	unknown dam2	male	tissue	case	palatoschisis, owner reported	unknown	unknown	unknown		-	+
SSC076	SSC040	unknown dam3	female	tissue	case	palatoschisis, owner reported	unknown	unknown			-	+
SSC079	SSC040	unknown dam4	female	tissue	case	palatoschisis, owner reported	unknown	unknown			-	+
SSC004	SSC040	unknown dam5	female	tissue	case	palatoschisis, owner reported	unknown	unknown			-	+
SSC029			female	blood	dam						-	-
SSC030			female	blood	dam						-	-
SSC031			female	blood	dam						-	-
SSC032			female	blood	dam						-	-
SSC033			female	blood	dam						-	-
SSC034			female	blood	dam						-	-
SSC035			male	blood	potential sire						-	-
SSC036			male	blood	potential sire						-	-
SSC037			male	blood	potential sire						-	-
SSC038			male	blood	potential sire						-	-
SSC039			male	blood	potential sire						-	-
SSC040			male	blood	confirmed sire						+	+
