# Supplementary material for: Chromosomal imbalance in pigs showing a syndromic form of cleft palate
Source: BMC Genomics. 2019 May 8;20:349. doi: 10.1186/s12864-019-5711-4 (PMC6505205; doi:10.1186/s12864-019-5711-4)
Supplement: Supplementary file 2 — Primers used for genotyping. (PDF 153 kb) [file 12864_2019_5711_MOESM2_ESM.pdf]

Additional file 2 Primers used for genotyping

Primer	Sequence	Product [bp]	SScrofa11.1 position start	SScrofa11.1 position stop
F8	AGGGCCACACAGTTGTCAAA	1833	25854091	25854110
R8	CCATCTACTGATGCTTTGTTTTTCA		25855899	25855923
F14	CCCAGTACCTGACCCCAGTA	1694	109708647	109708666
R14	CATCACTTTTTCTGAGTGAGTCCA		109710317	109710340
