# Supplementary figures and images for: Chromosomal imbalance in pigs showing a syndromic form of cleft palate
Source: BMC Genomics. 2019 May 8;20:349. doi: 10.1186/s12864-019-5711-4 (PMC6505205; doi:10.1186/s12864-019-5711-4)

SSC003

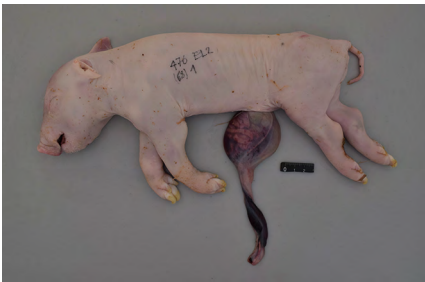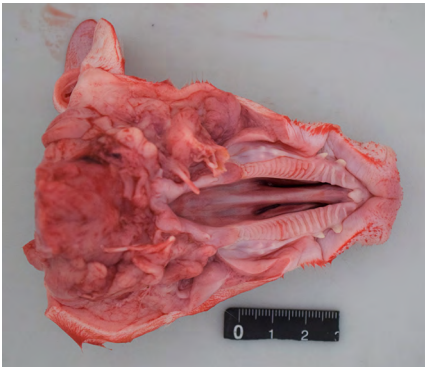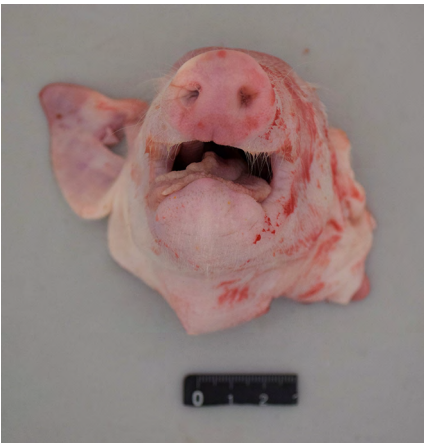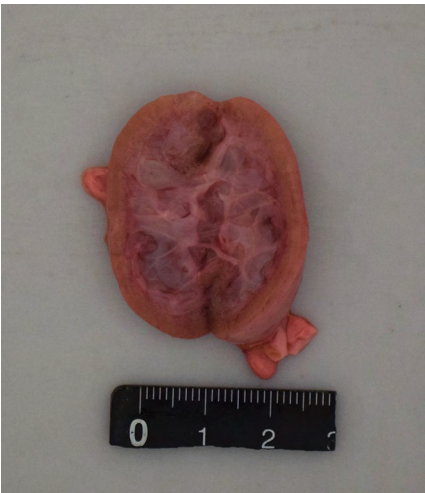

SSC006

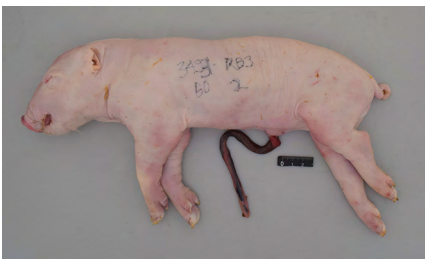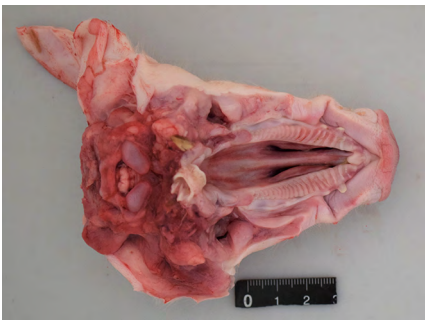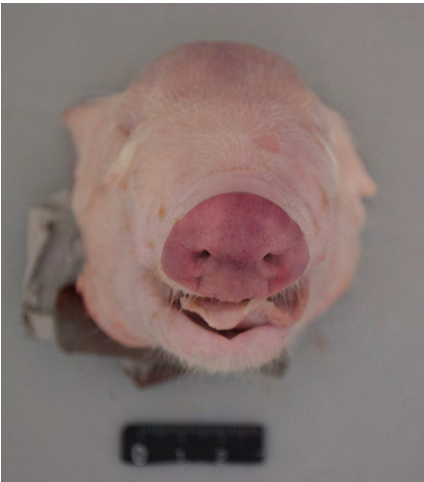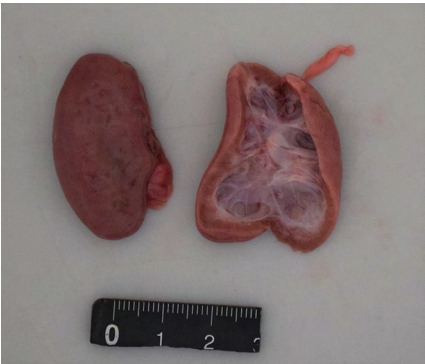

SSC007

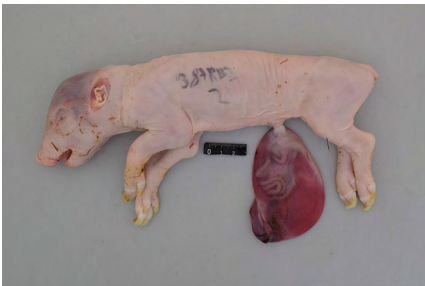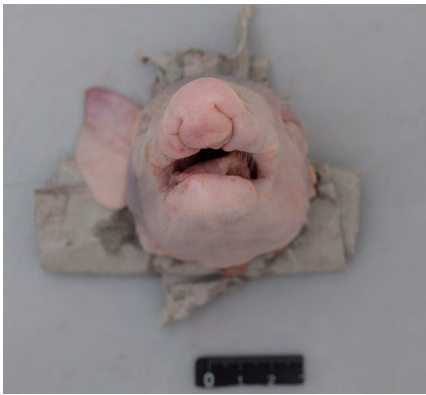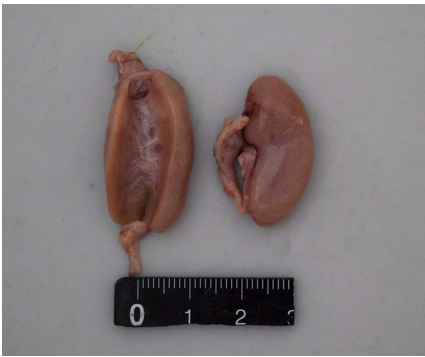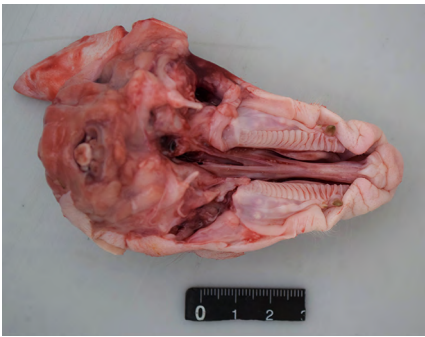

SSC008

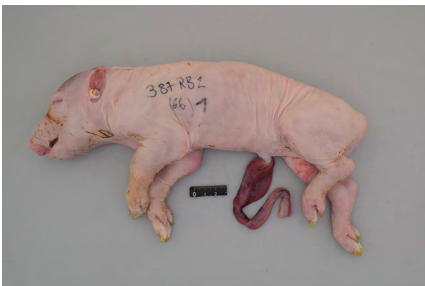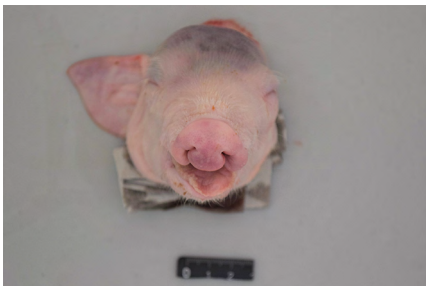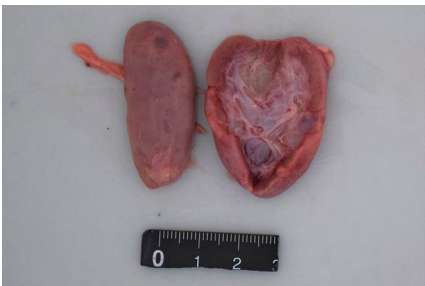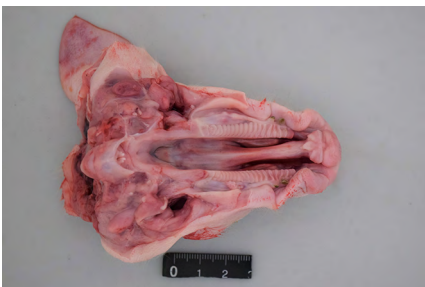

SSC009

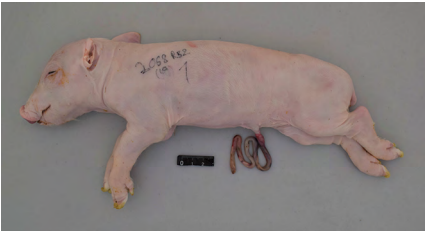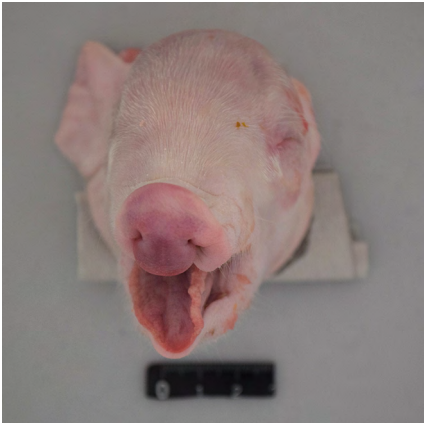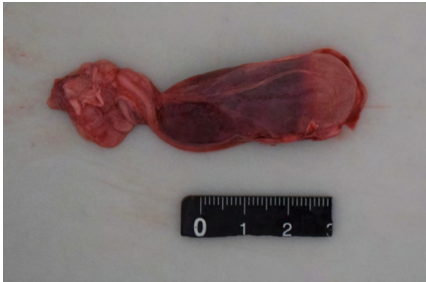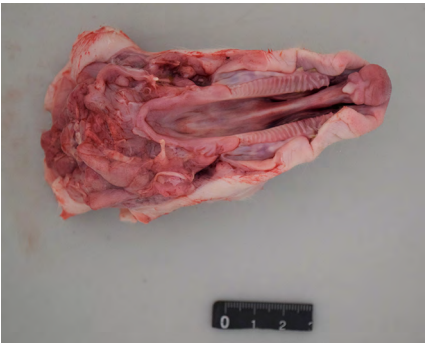

SSC0010

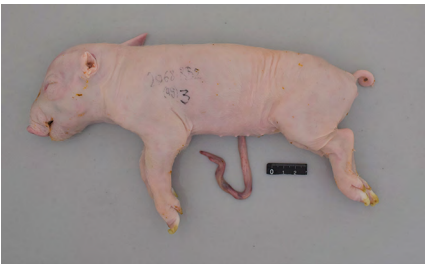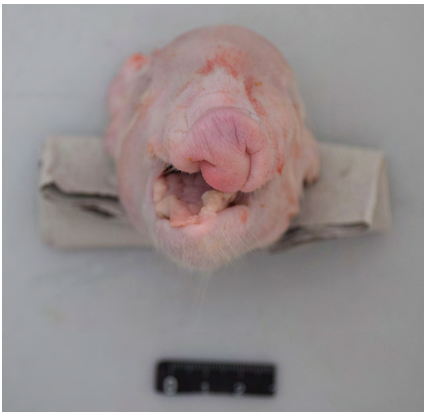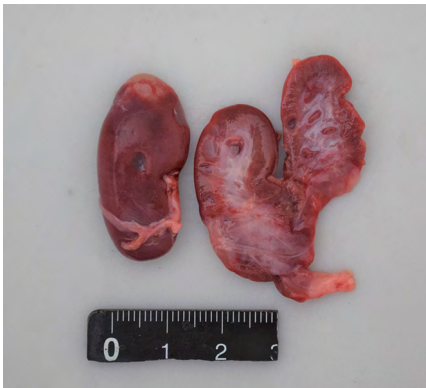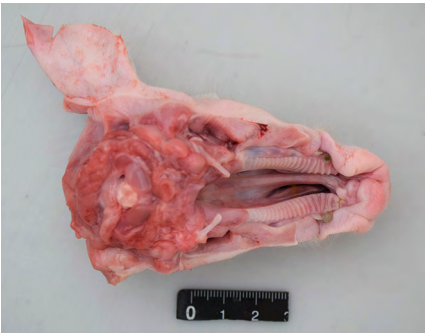

SSC011

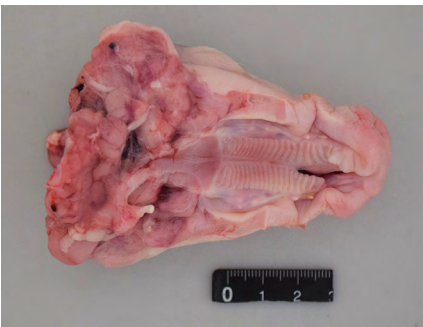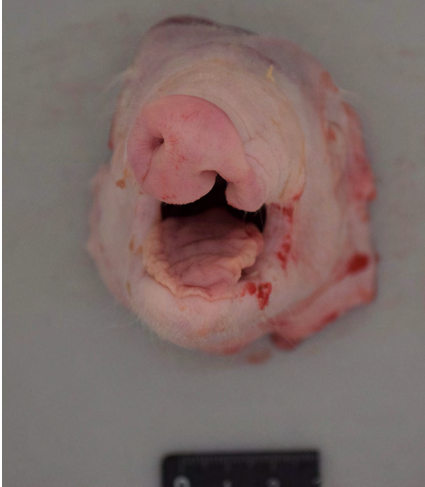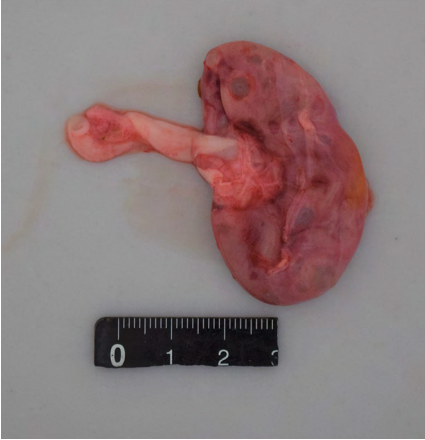

SSC012

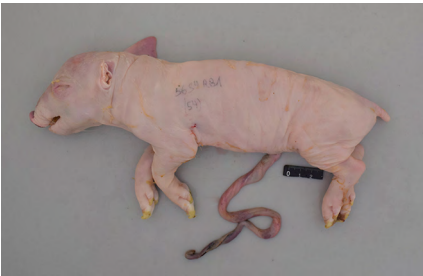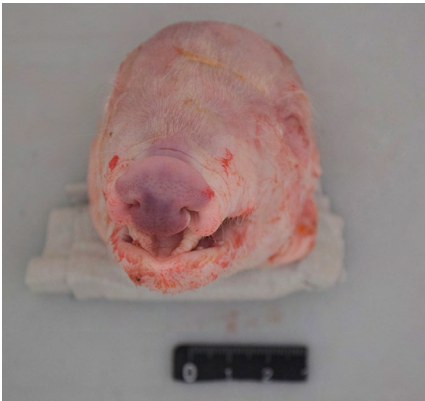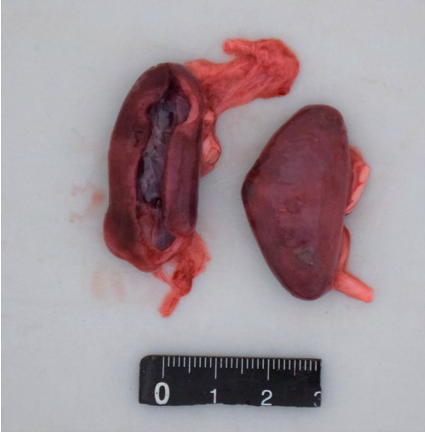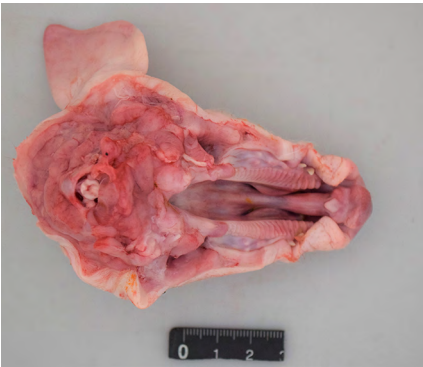

SSC013

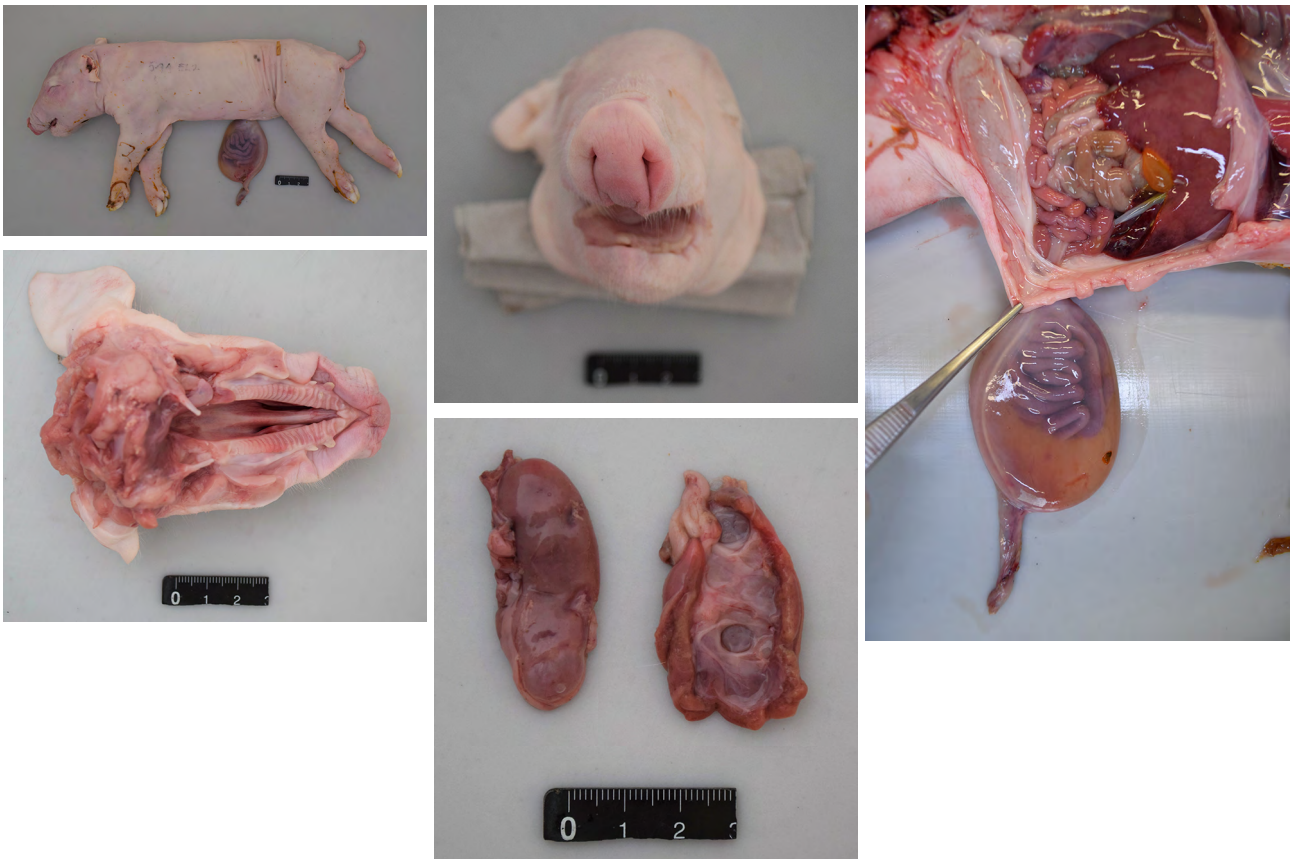

SSC014

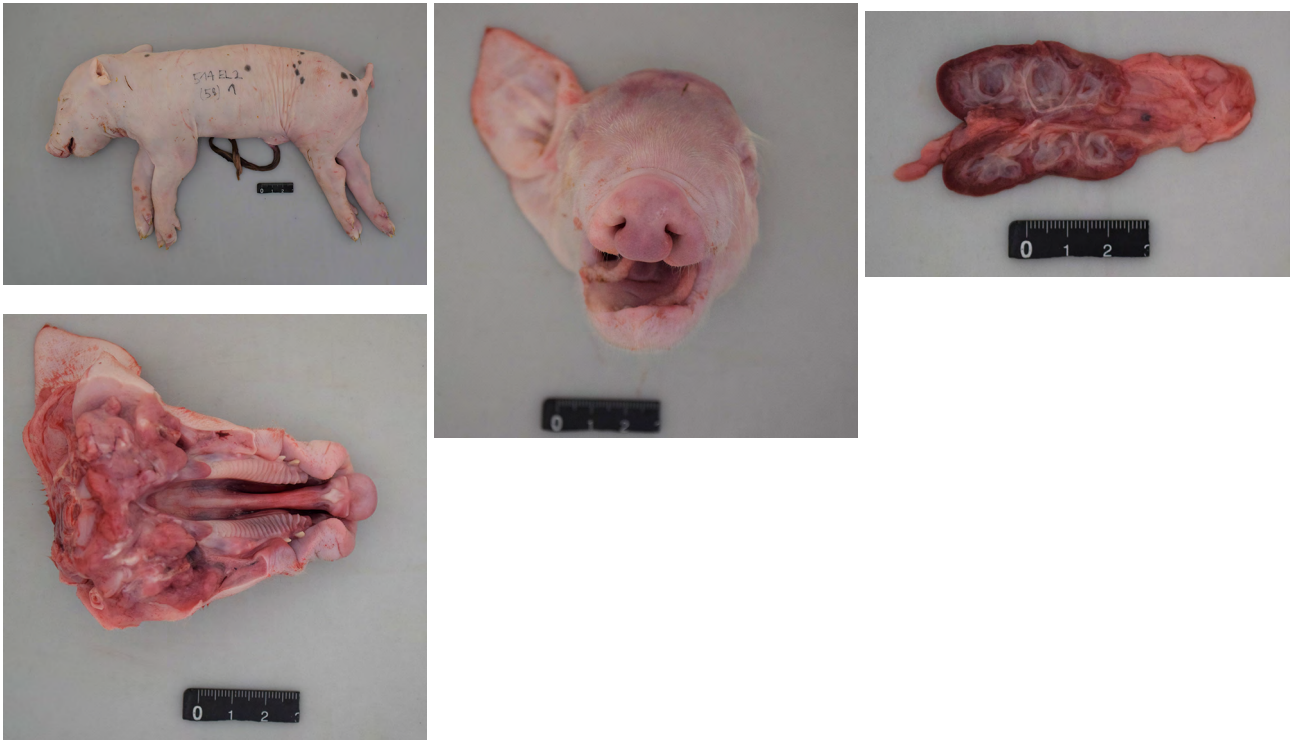

SSC015

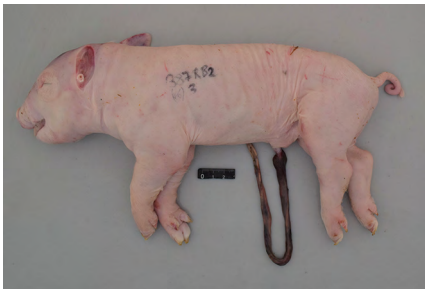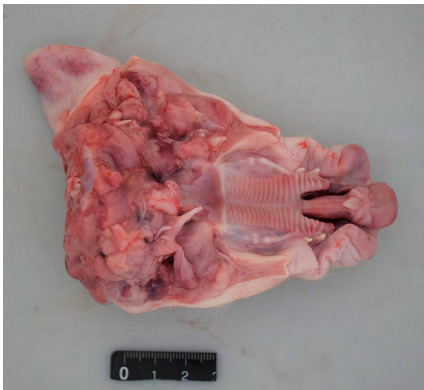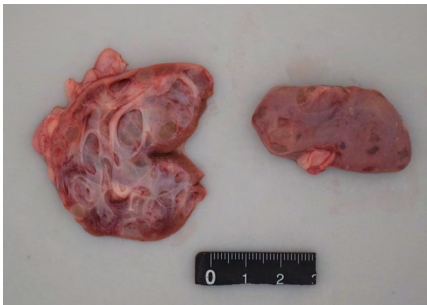

SSC016

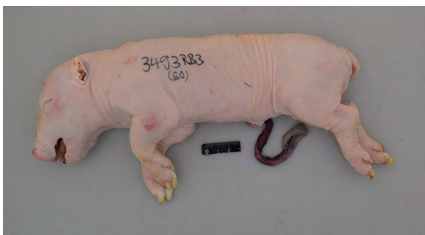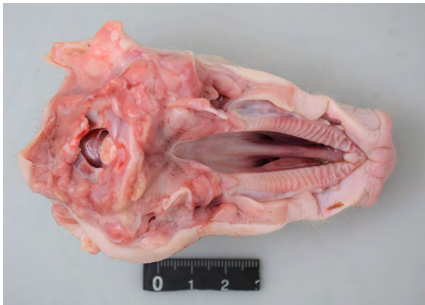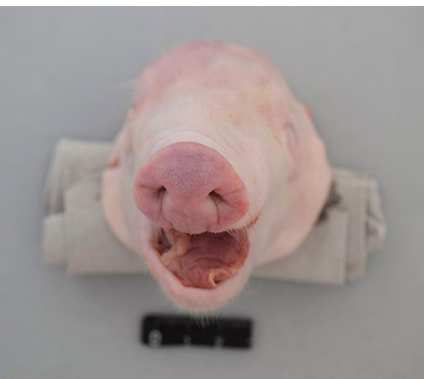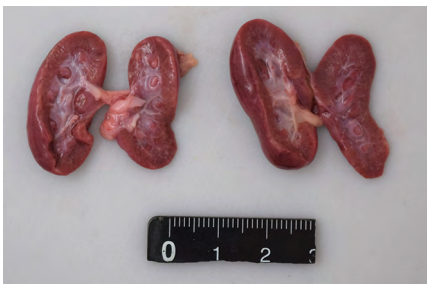

Supplement: Supplementary file 3 — Detailed phenotype records of 12 affected piglets. (PDF 3111 kb) [file 12864_2019_5711_MOESM3_ESM.pdf]

**A**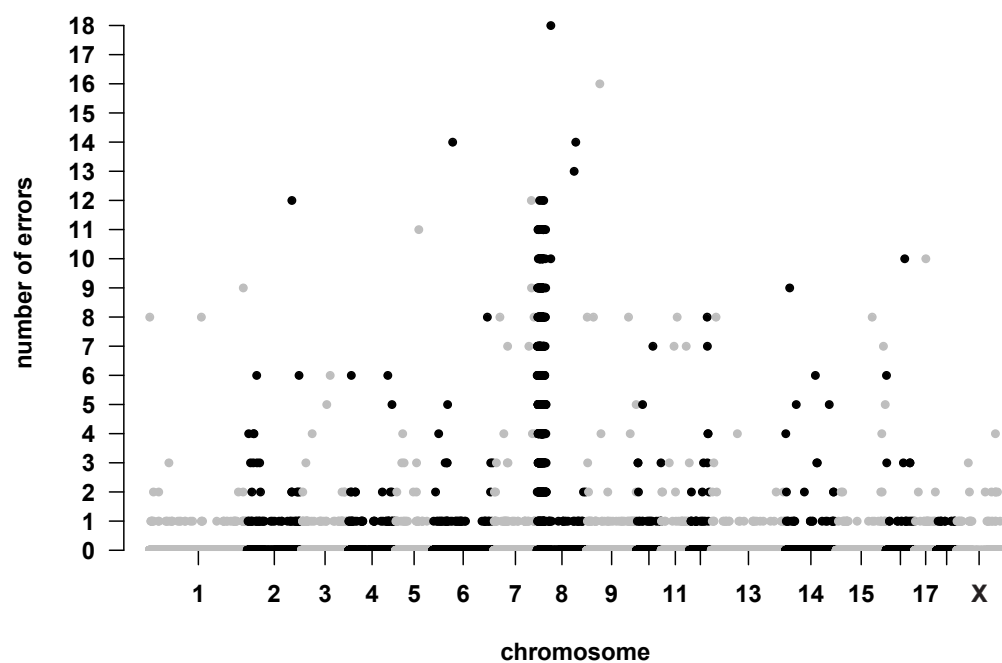**B**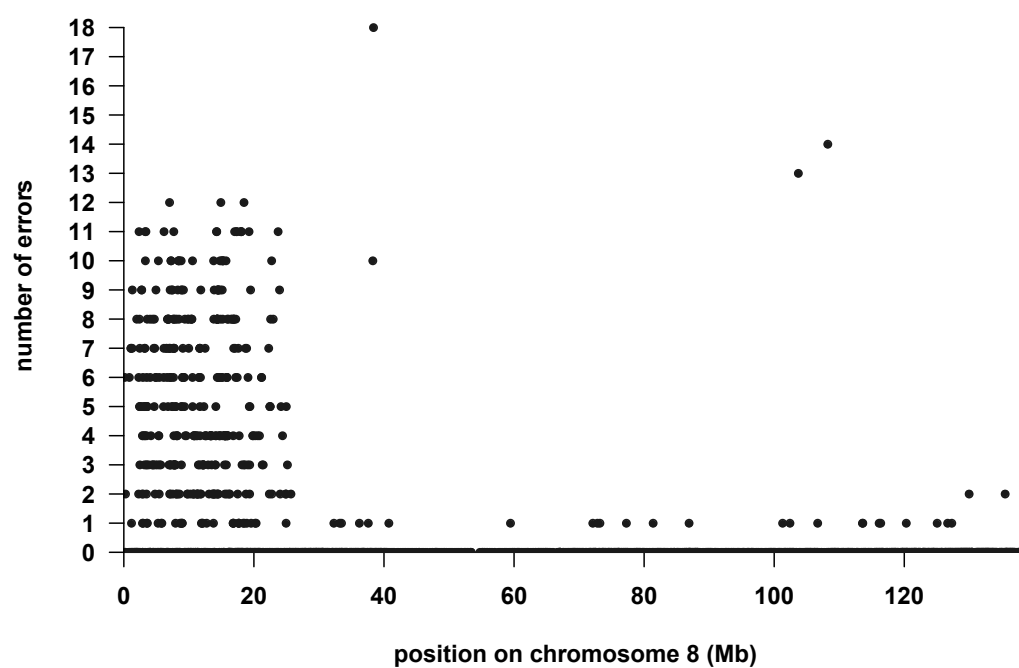

Supplement: Supplementary file 4 — Mendelian error mapping. The genomic position of the 3178 SNVs showing Mendelian errors is shown along the porcine chromosomes (above). Note the clustering of the errors in the proximal 25 Mb of chromosome 8 (below). (PDF 1105 kb) [file 12864_2019_5711_MOESM4_ESM.pdf]

Chromosome 8

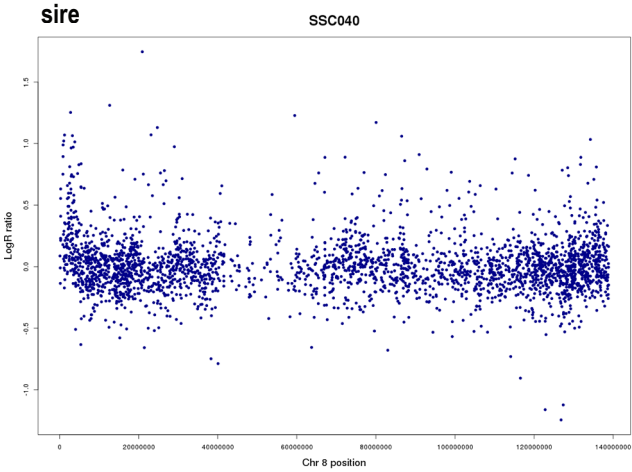

Chromosome 14

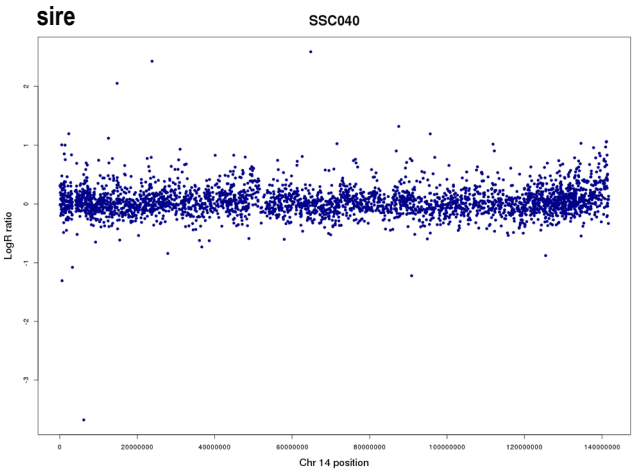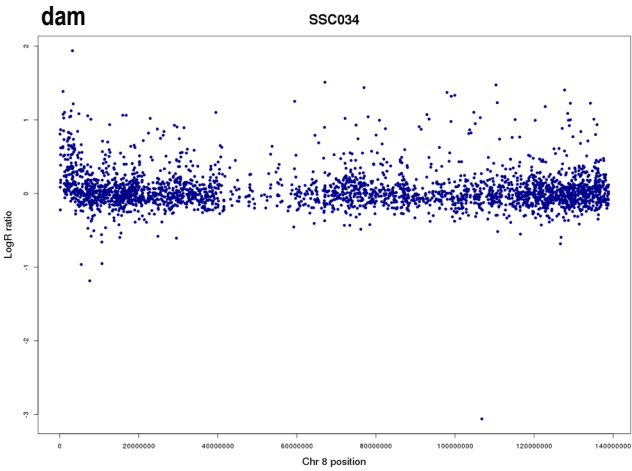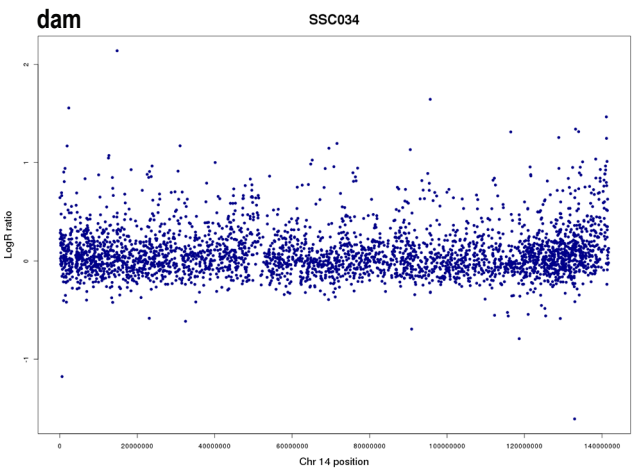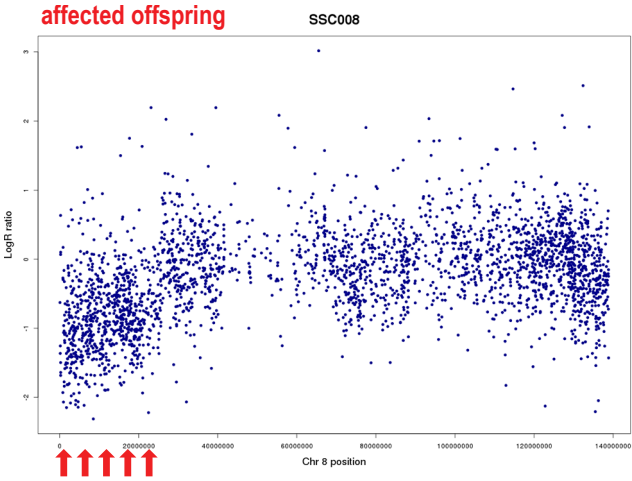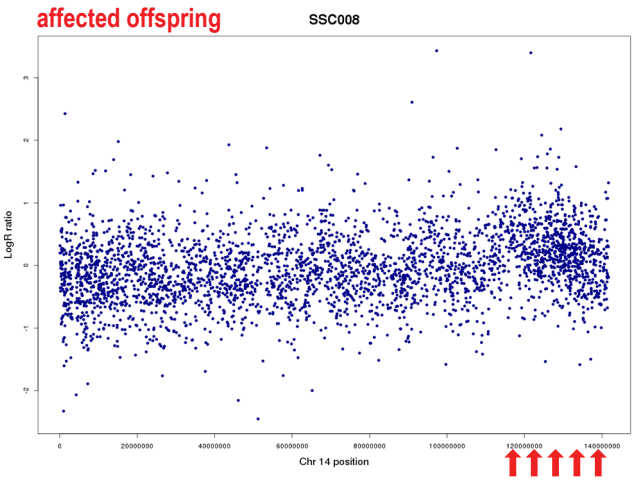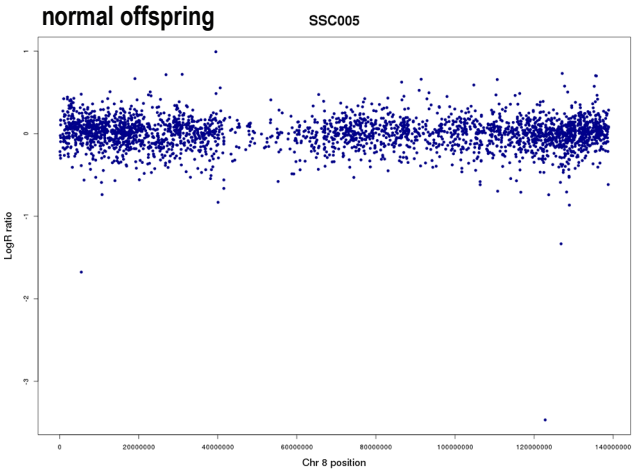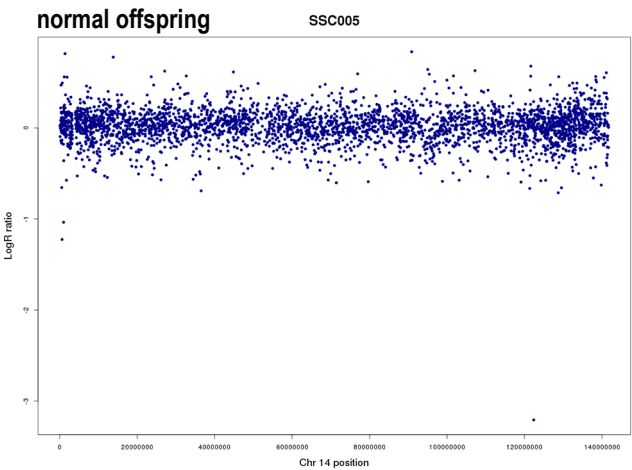

Supplement: Supplementary file 5 — Examples of log R ratio (LRR) plots. The LRR is shown along the chromosomes for all SNVs on the array. Examples for 4 animals are shown for chromosomes 8 and 14. Note that the LRR drop in the proximal region of chromosome 8 and the increase in the distal part of chromosome 14 in the affected piglet (shown in red). (PDF 2021 kb) [file 12864_2019_5711_MOESM5_ESM.pdf]

## Litter SSC029

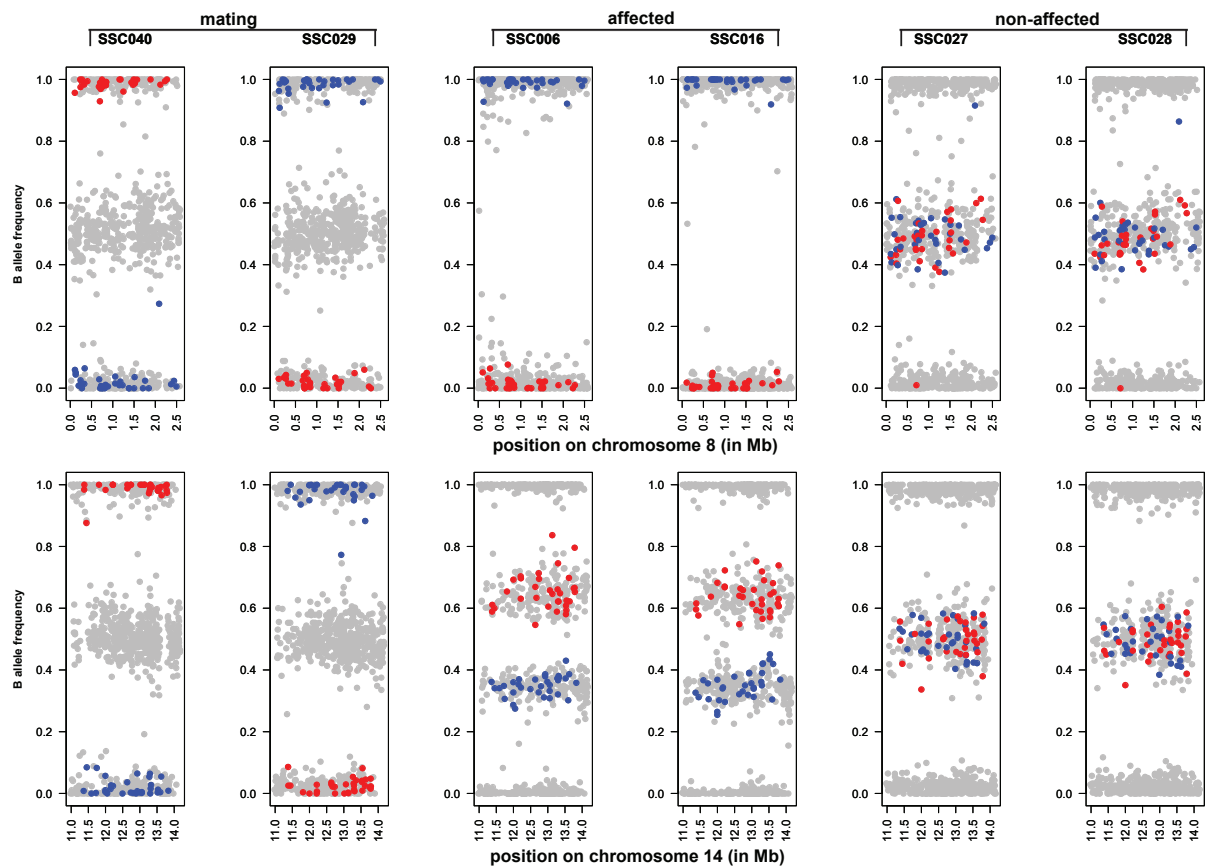

## Litter SSC030

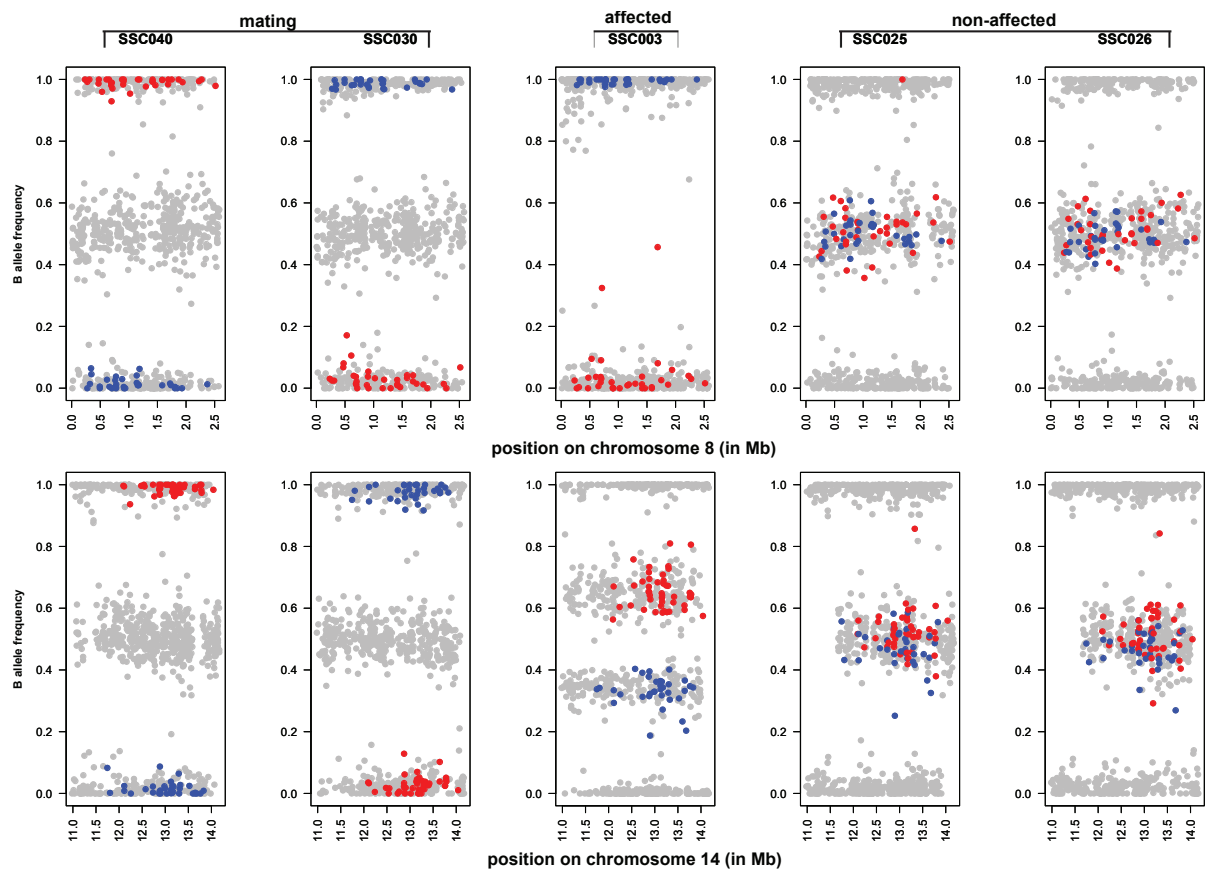

Litter SSC031

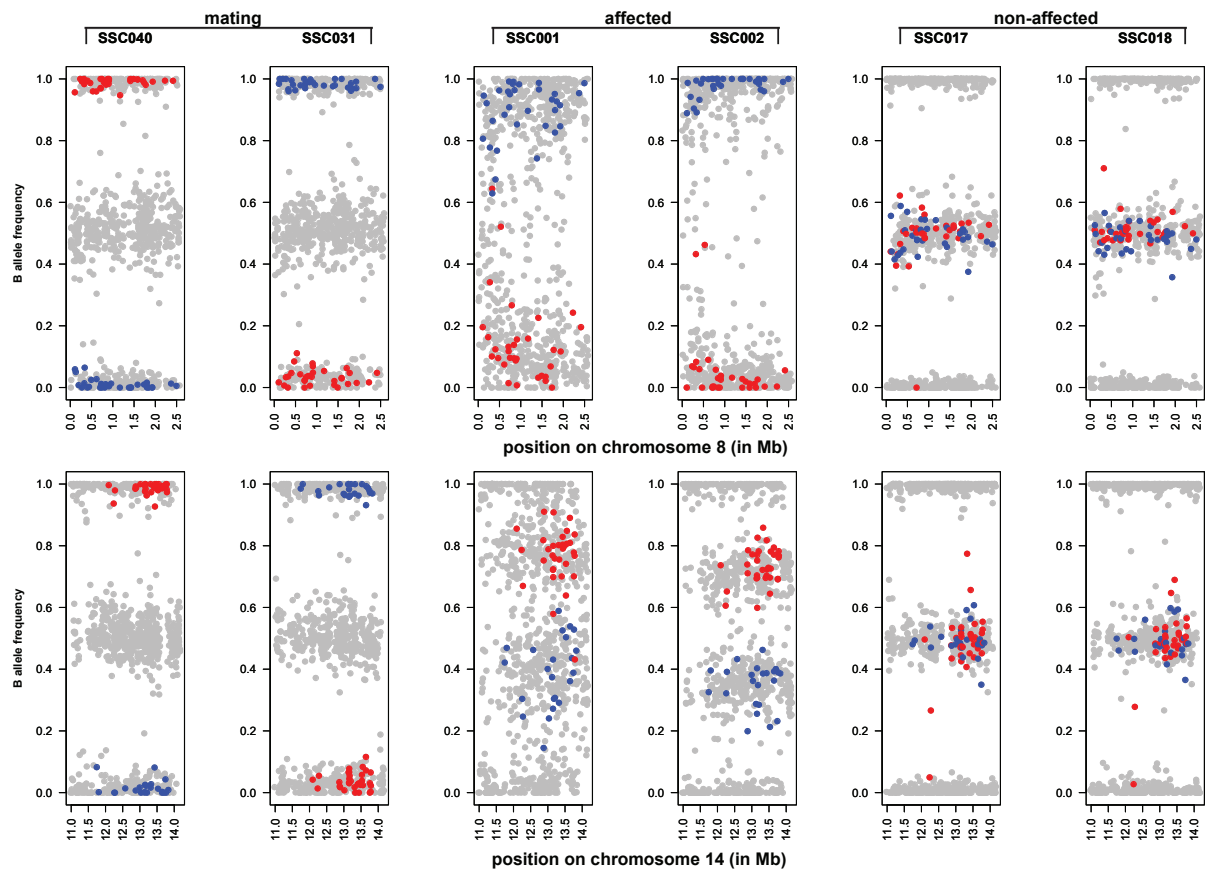

Litter SSC032

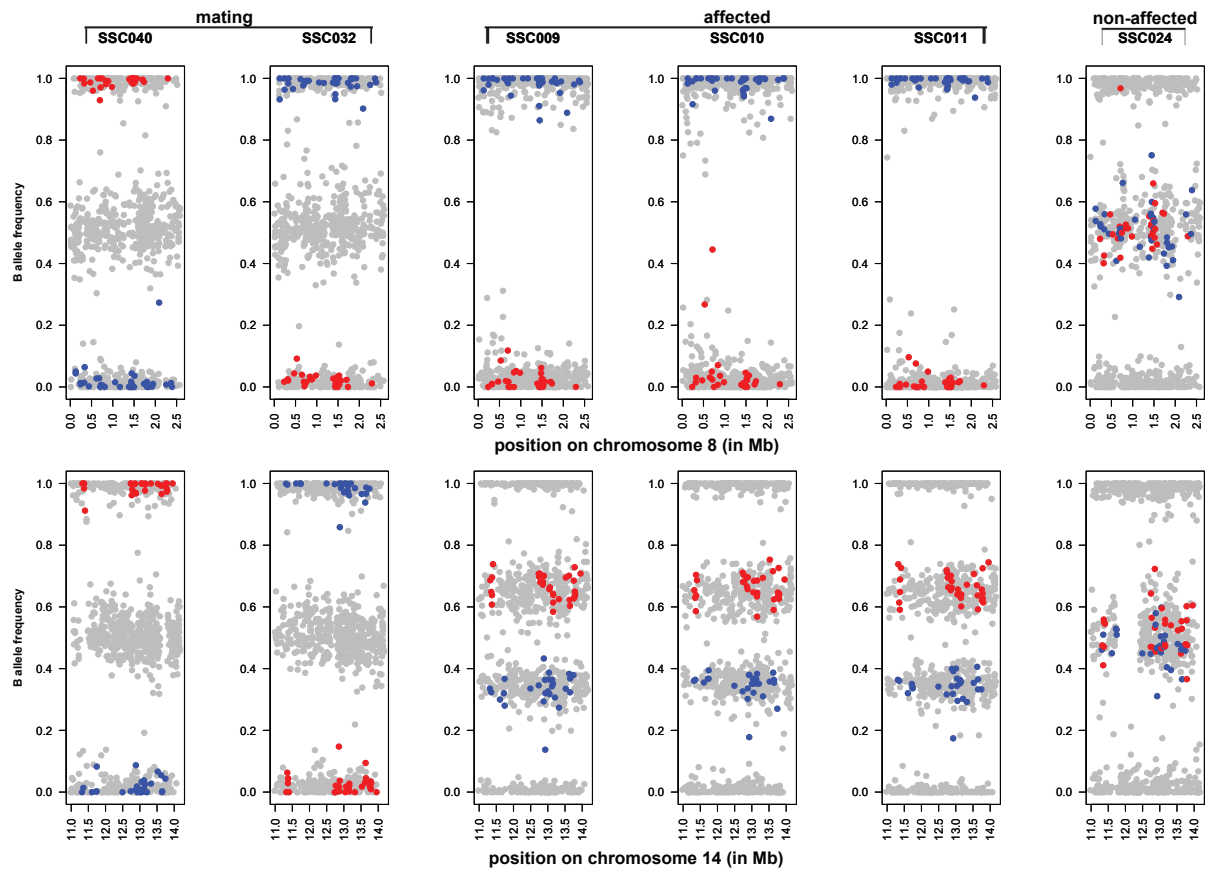

## Litter SSC033

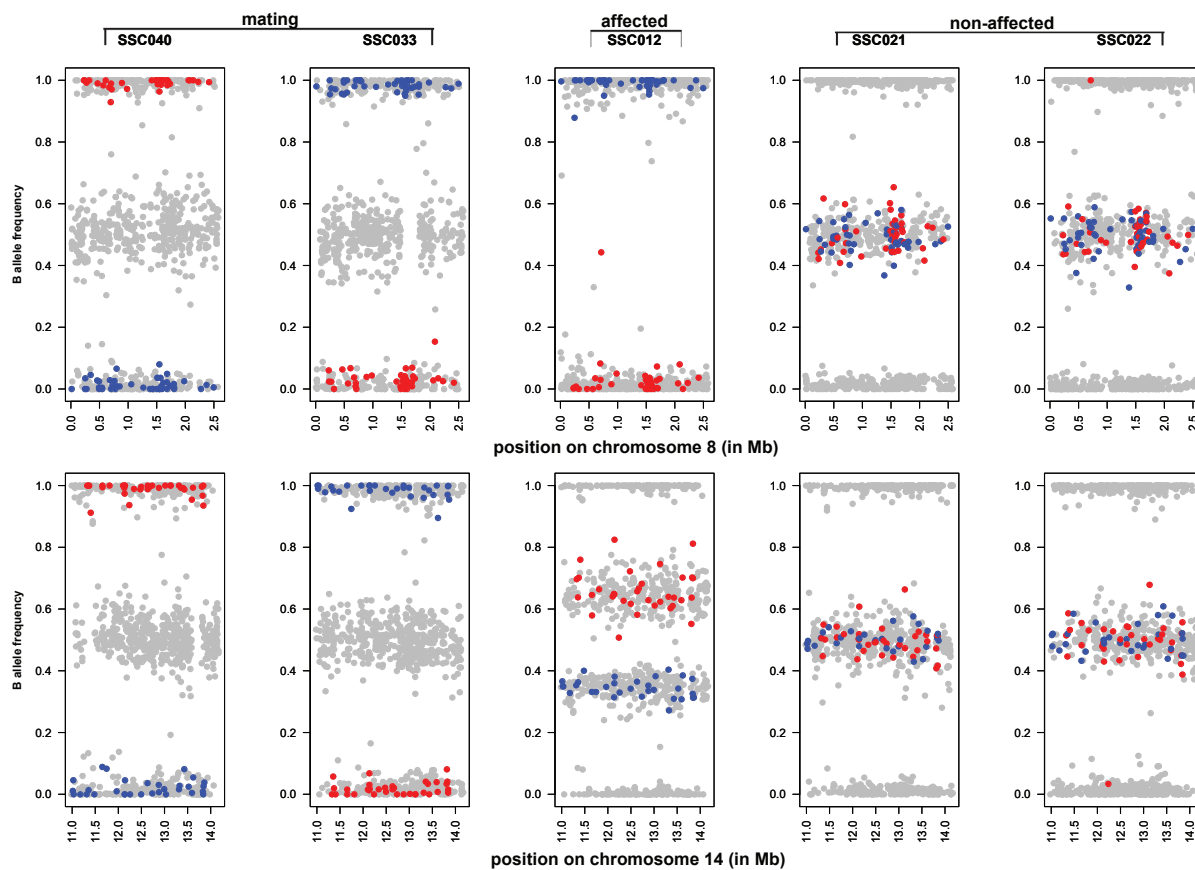

## Litter SSC034

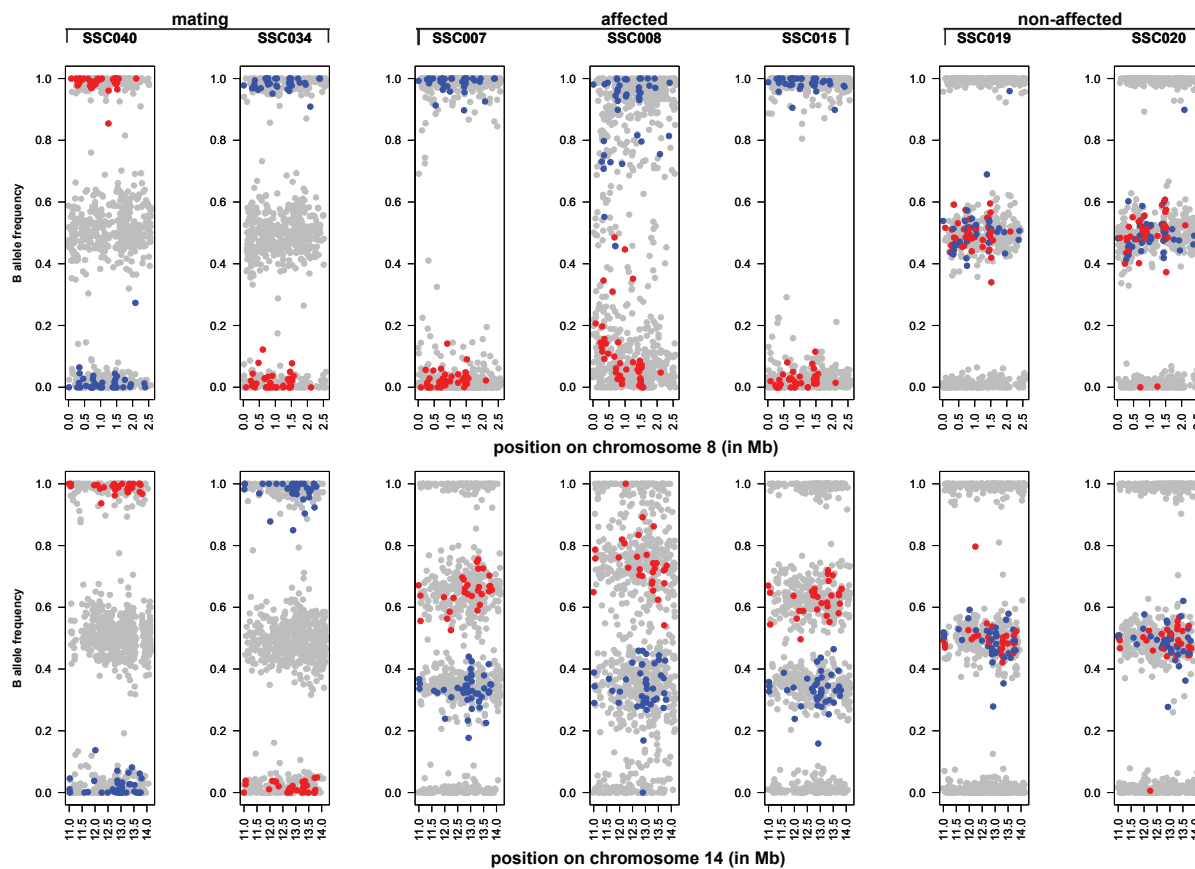

Supplement: Supplementary file 6 — Segregation of SNV alleles. In all 6 examined families for both parents alternative homozygous SNVs (red/blue) were selected to determine their inheritance pattern. Note that in all affected piglets paternal homozygous SNVs show an underrepresentation on chromosome 8 and an overrepresentation on chromosome 14. For SNVs where the dams were homozygous for the alternative allele, an opposite segregation could be observed. (PDF 2183 kb) [file 12864_2019_5711_MOESM6_ESM.pdf]

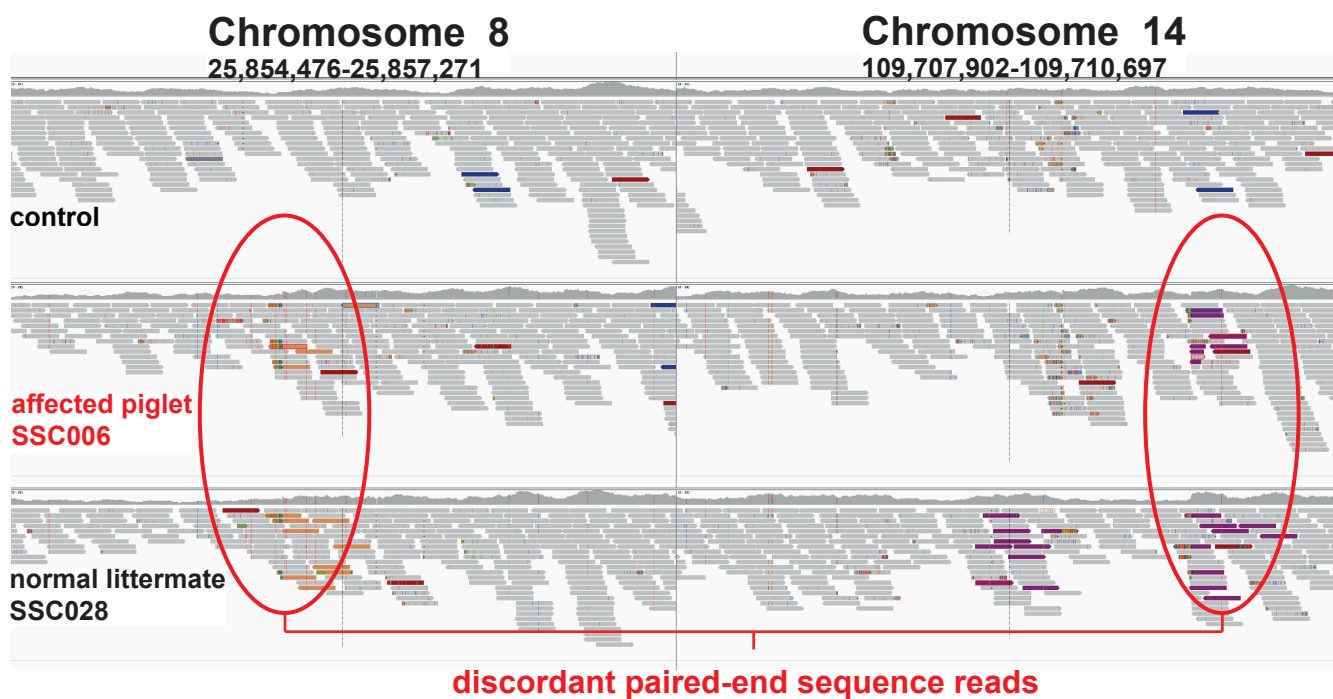

Supplement: Supplementary file 7 — IGV snapshot indicating the translocation. Note the reduced coverage on chromosome 8 and the increased coverage on chromosome 14 in the affected piglet. Paired-end sequence reads mapping on two different chromosomes are displayed in different colors. (PDF 2757 kb) [file 12864_2019_5711_MOESM7_ESM.pdf]

**A**

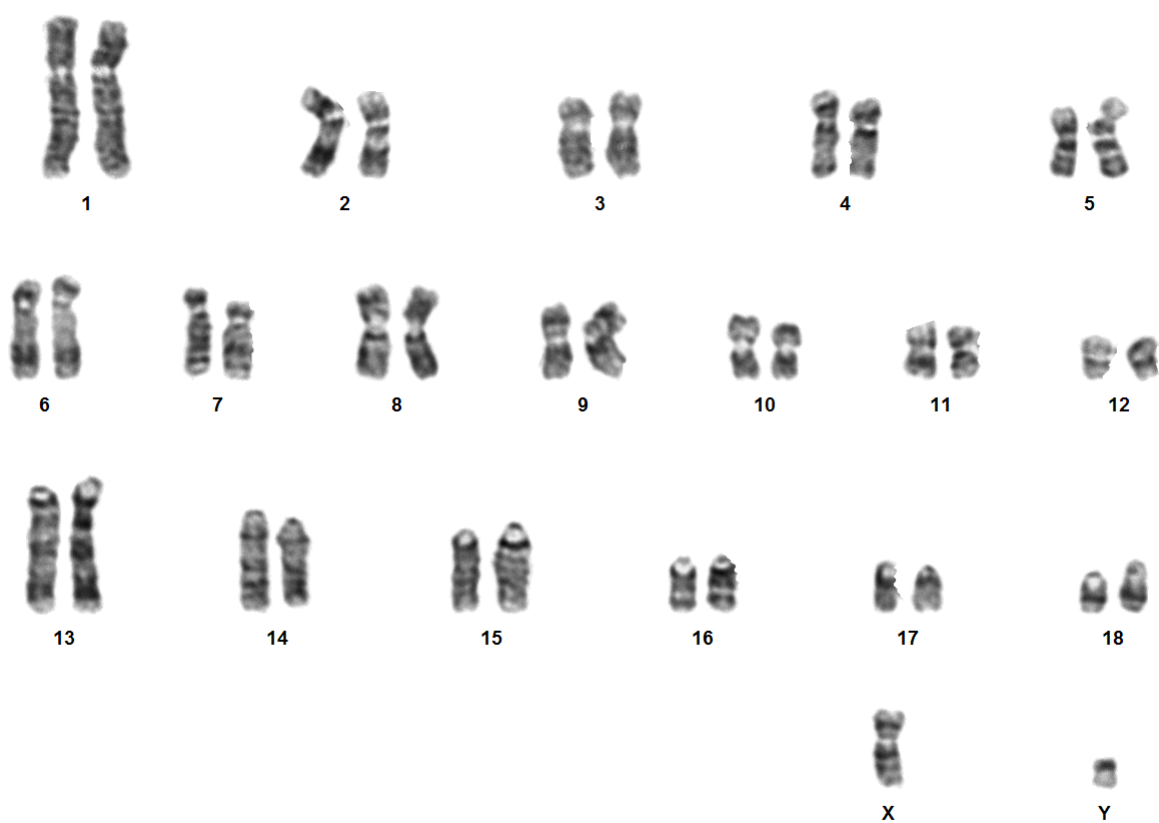

**B**

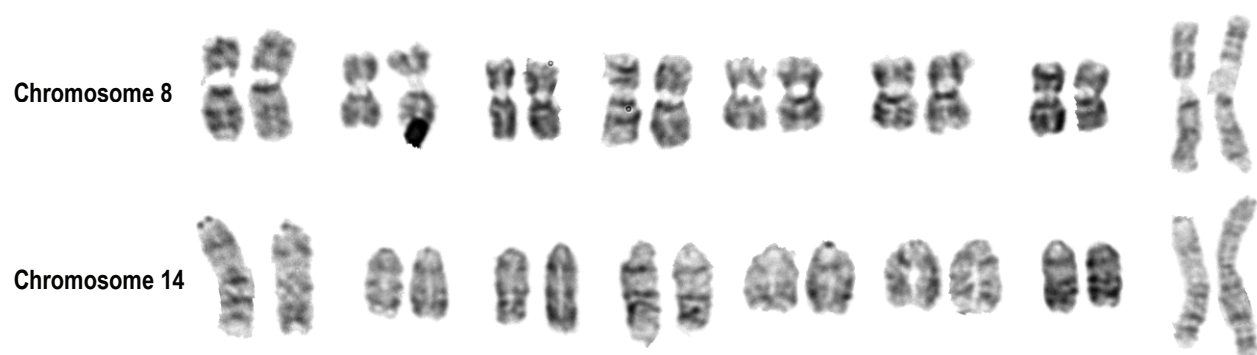

Supplement: Supplementary file 8 — Karyotype of the boar: 38, XY, t (8,14). Note that the two affected chromosomes show an unobvious abnormal banding profile (above). A comparison of the chromosomes 8 (top) and 14 (bottom) from 8 different cells of the boar revealed no microscopically visible difference (below). (PDF 1136 kb) [file 12864_2019_5711_MOESM8_ESM.pdf]
